# Hydrothermal conversion of toilet waste: effect of processing conditions on gas phase emissions

**DOI:** 10.1016/j.heliyon.2022.e09708

**Published:** 2022-06-13

**Authors:** Gerty J.H.P. Gielen, John P. Andrews, Christine M. Karbiwnyk, Mark J.C. Riddell, Sean W. Husheer, Daniel J. Gapes

**Affiliations:** Scion, Titokorangi Drive, Private Bag 3020, Rotorua 3046, New Zealand

**Keywords:** Hydrothermal, Toilet waste, Wet oxidation, Volatile organic compounds, Carbon monoxide, Decentralised sanitation

## Abstract

Globally, many populations suffer from a lack of access to basic sanitation facilities. This is partly caused by a combination of water resource shortages and the high cost of conventional centralised treatment systems. A novel decentralised treatment technology based on sub-critical hydrothermal processing of organic wastes at toilet-scale, contributes to addressing these economic and resource limitations. To be effective, this technology needs to satisfy a broad range of environmental and safety considerations, including the nature and quantity of formed gas products. We investigated the impact of four process parameters (temperature; O_2_: COD ratio (λ); time; feed solids content) on off-gas composition by quantifying volatile organic compounds (VOCs), CO, H_2_ and CO_2_ in factorial experiments. Temperature and λ influenced VOCs generation greatly. The lowest VOC emissions occurred at 200% λ and 300 °C. Aldehydes and ketones were mostly generated at 200% λ and intermediate temperatures, sulphur compounds in the absence of oxygen, and aromatics, furans, and pyrroles at intermediate oxygen levels and elevated temperatures. Most CO was created at 300 °C but its concentration decreased at longer processing times. Processing conditions have complex impacts and require careful consideration when designing for real world deployment.

## Graphical abstract

**Figure:**
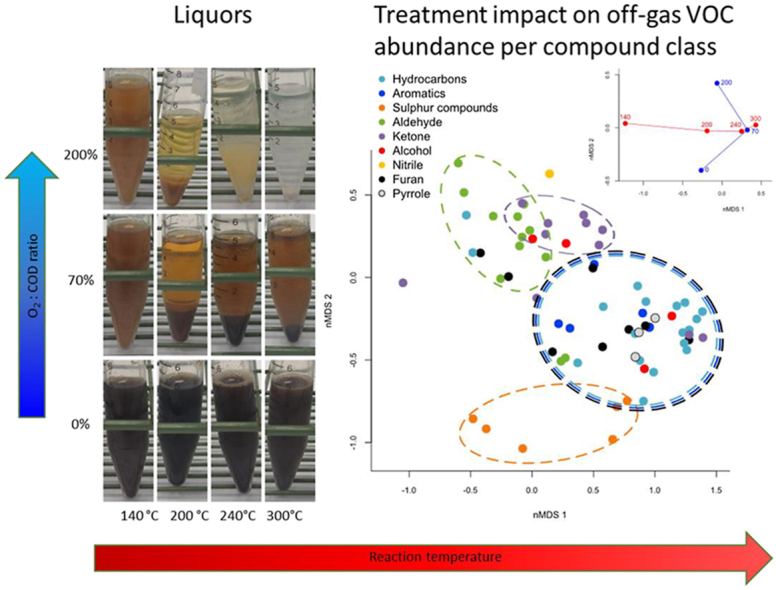

## 1 Introduction

Safe and affordable sanitation, the management of human excreta, is foundational to a healthy and dignified life. Unfortunately, this ideal, articulated in the UN Sustainable Development Goal 6 – “Ensure availability and sustainable management of water and sanitation for all” – remains inaccessible to a large proportion of the global population (Hii et al., 2014; World Health Organization and United Nations Children's Fund, 2020). Indeed, the world is “alarmingly off-track to deliver sanitation for all by 2030”, with over half of the world's population (4.2 billion people) using inadequate sanitation like open defecation and other untreated disposal to land or water (World Health Organization and United Nations Children's Fund, 2020). A clear and urgent need exists to develop a sanitation value chain– from the toilet user through to treatment, resource recovery and final disposal of treated materials. Hydrothermal processing (HP) is a promising technology for application into a sanitation value chain by treating household toilet waste. The HP technology is based around the thermochemical conversion of wet organic materials like toilet waste or sewage sludge, via the application of elevated temperatures and pressures either in the absence of oxygen (i.e. thermal hydrolysis) or in the presence of oxygen (i.e. wet oxidation) (Yousefifar et al., 2017a). An advantage of HP technology is that higher strength organic wastes can be processed in wet or slurry form, without the need to remove water in a pre-treatment step. This can be especially beneficial for organic waste streams that are otherwise difficult to treat because they contain too much water to process as a solid and too many solids to process as a liquid. Other advantages of this technology include comprehensive pathogen destruction and sterilisation; very high rates of organic pollutant degradation compared with biological systems; extensive reduction in mass of particulate organic solids (>90%); improved dewaterability of residuals; potential for auto-thermal processing under oxidative conditions; and potential recovery of valuable resources from the liquor such as ammonia, phosphate, and short chain fatty acids (from oxidative processes), or biocrude and hydro-char (from non-oxidative processes) (Andrews et al., 2015; Baroutian et al., 2016; Conti et al., 2020; Hii et al., 2014; Munir et al., 2018).

Currently, HP technology for waste treatment is mostly employed at large sewage treatment plant and industrial scale (Debellefontaine and Foussard, 2000; Hii et al., 2014) and usually in continuous processing mode. Our work has been to down-scale HP technology, matching toilet waste loads to small batch processing reactors, and aiming for safe and effective operation at the applied elevated pressures and temperatures. Success here supports the viability of HP technology as a decentralised alternative to centralised sewage treatment and to traditional disposal options. The composition of solid and liquid products after HP of sewage sludge under various process conditions has been reviewed in detail by Suárez-Iglesias et al. (2017). They indicated that increased processing temperatures led to increased solubilisation of biomolecules, greater decomposition of proteins and carbohydrates, and greater yields for carboxylic acids like acetic acid. They also highlighted the potential for recovery of valuable compounds from sewage sludge when combined with post-treatments. Hydrothermal processing can be applied to a diverse range of wet organic materials for a variety of applications such as domestic sewage, biosolids, pharmaceuticals, and agricultural wastes for waste treatment, pretreatment of lignocellulosic feedstocks to enhance enzymatic degradation, pretreatment of sewage sludge to improve anaerobic digestion, hydrothermal carbonization of biomass for production of hydro-char and biofuel, and hydrothermal processing of inorganics for nanoparticle synthesis (Debellefontaine and Foussard, 2000; Hii et al., 2014; Ruiz et al., 2020; Sharma et al., 2020; Yang and Park, 2019; Yousefifar et al., 2017b; Zeng et al., 2018; Zhan et al., 2020). For the decentralised treatment of household toilet waste, HP is particularly an attractive technology because of the short processing times at high temperatures, the considerable reductions in waste solids and pathogens and the potential for resource recovery.

The HP treatment technology encompasses a range of related processes. These are distinguished by their range of reaction conditions, particularly process temperatures and pressures, in relation to the critical point of water (374 °C, 22.1 MPa), and the oxidative state of the system. The processes are described by representative terminology including thermal hydrolysis, hydrothermal liquefaction, hydrothermal carbonisation, hydrothermal gasification, wet oxidation, wet air oxidation, sub-critical water oxidation and supercritical water oxidation. In this study, we focused on sub-critical hydrothermal processing that occurs below the critical point of water, typically at temperatures between 150 °C–370 °C, pressures between 20 bar–350 bar and residence times between 2 min–120 min (Prince-Pike et al., 2015). Process parameters investigated in this study were chosen to cover representative conditions for HP processing under non-oxidative thermal hydrolysis and sub-critical wet oxidation processes (Munir et al., 2018). Our overarching aim was the development of a novel, affordable, and efficient decentralised household scale system for treating toilet waste based on HP technology.

Challenges exist for the HP technology – a high temperature and pressure reaction requires an appropriate reactor specification, and this must be fitted with high quality safety measures. Ultimately, this will lead to affordability challenges. On the process side, the nature of the gaseous emissions from these processes is poorly described. Given the close proximity of people to the potential technology application, safe deployment demands a significant knowledge improvement in that area. The novelty of this study lies in an improved understanding of the process conditions that influence the volatile and gaseous reaction products. This is necessary so that HP treatment can be designed to have minimal harmful gas emissions to the surrounding air. Our study was completed to expand the knowledge about volatile emissions from hydrothermal processing of organic waste biomass.

Increasing combustion of carbon containing fuels over the last century have been responsible for the progressive increase in air pollutants like carbon dioxide (CO_2_), carbon monoxide (CO) and volatile organic compounds (VOC) (Kampa and Castanas, 2008). The gas stream from wet oxidation of chemical industry wastes, sewage sludges and toilet wastes have been reported to contain between 0.5% and 25% of carbon monoxide (CO) and volatile organic compounds (VOC) together (Anthony and Hintze, 2014; Debellefontaine and Foussard, 2000). A study specifically treating urine/faeces mixtures at 400 °C, reported that CO concentrations peaked at 10% in the depressurised gas (NASA and MODAR, 1983). Andreae and Merlet (2001) compared emissions of CO_2_, CO and pyrogenic organic compounds across a range of biomass burning locations (grasslands and forests) and sources (biofuel, charcoal and agricultural), indicating that CO_2_ was emitted the most, followed by CO and a range of VOC compounds. Exposure to both CO_2_ and CO can have health impacts and cause asphyxiation although CO is more toxic at lower concentrations and CO exposure can have serious health impacts such as neuropsychological impairment, respiratory issues, and reduced birth weights (Kampa and Castanas, 2008; Stieb et al., 2012). In addition, both CO_2_ and CO contribute to global warming; CO_2_ directly and CO as a precursor to ozone (Majstorović et al., 2020; Rogelj et al., 2016). This not only shows the need to minimise emissions of CO_2_ and CO to the atmosphere for environmental protection reasons but also the need to avoid CO emissions in the indoor air environment for human health reasons.

Due to the volatile nature of VOCs, upon formation and without further treatment or containment, they are likely released into the air. Then, depending on composition, yield and exposure, they could potentially have adverse health effects such as eye, skin, mucous membrane irritation, and respiratory tract irritation, chronic illness such as asthma, obstructive pulmonary disease, and cancer (Du et al., 2014). A range of VOCs have been measured in many different environments such as indoor (Vu et al., 2019), outdoors (Ramírez et al., 2012), industrial emissions (Chang et al., 2019), wastewater treatment plants (Widiana et al., 2019) and cooking fumes (Zhang et al., 2019). Typical VOCs of concern in off-gas include aldehydes (acetaldehyde, pentanal, hexanal, heptanal), acroleins, benzene, 1,3-dimethylbenzene, toluene, m-xylene and dimethyl disulfide. Aromatics like benzene, toluene, ethylbenzene are carcinogens while aldehydes contribute to a range of mutagenic and cytotoxic mechanisms (O'Brien et al., 2005).

The objective of this study was to determine the effect of temperature, time, oxygen and solids content, on the off-gas composition by quantifying the production of volatile organic compounds, CO and CO_2_. Our research was framed around understanding how oxidative state impacts the nature and yield of VOC emissions. Specifically, our main hypothesis was that, within a hydrothermal processing system, a limiting oxygen supply produces more CO and VOCs as a final carbon sink for the organic carbon, than when plenty of oxygen is available.

## 2 Materials and methods

### 2.1 Materials

Two samples of toilet waste with different solids contents were prepared as bulk feedstock for the experiments. The high solids samples (1) comprised of 16 wt% human faeces, 2 wt% toilet paper, 82 wt% urine and no water, and the low solids sample (2) of 7 wt% human faeces, 1 wt% toilet paper, 75 wt% urine and 18 wt% water. These samples were each homogenised for 10 min at 25,000 rpm (GLH 850, Omni International). Homogenised samples were sub-divided into multiple syringes and stored in a freezer at -20 °C. The composition of the homogenised feedstocks were determined according to standard methods (Baird and L., 2017). The high solids feedstock (1) was characterised as pH 9.9, total solids (TS) 76,772 mg/L, volatile solids (VS) 60,872 mg/L, total suspended solids (TSS) 56,112 mg/L, volatile suspended solids (VSS) 48,930 mg/L, total chemical oxygen demand (tCOD) 109,653 mg/L, total carbon (C) 35,400 mg/L and total nitrogen (N) 6,880 mg/L. The low solids feedstock (2) was characterised as pH 9.9, TS 30,892 mg/L, VS 20,914 mg/L, TSS 17,389 mg/L, VSS 15,098 mg/L, tCOD 37,592 mg/L, total C 14,200 mg/L, and total N 3,720 mg/L.

### 2.2 Hydrothermal treatment

Hydrothermal experiments were carried out in 15 mL tubular stainless-steel pressure reactors according to a full factorial design with 4 independent variables and a replicate of one. For each run, 5 g of feed material was put into reactors and then purged three times with either pure nitrogen or oxygen gas. An oxygen gas overpressure was added at three stoichiometric ratios relative to the chemical oxygen demand. This oxygen to fuel equivalence ratio (λ) is described in detail elsewhere (Hübner et al., 2016). In our experiment, the chosen O_2_:COD ratio (λ) was either 0% (non-oxidative, only nitrogen), 70% and 200%. The reactors were immersed into pre-heated, fluidising sand and mechanically shaken to mix the liquid and gas phase. After each experiment, the closed reactor was lifted out of the hot sand and immediately cooled to room temperature, the off-gas captured in a Tedlar bag and the liquor analysed for total chemical oxygen demand (COD). Heat-up and cool down times depended on target temperatures but were less than 5 min for all treatments.

The final gas volume was determined based on Boyle's gas law, whereby a volume of enclosed gas assuming a constant temperature varies inversely with the gas pressure. Therefore, the product of the final pressure contained within the known reactor headspace volume (15 mL) measured after each run but before gas sampling was equal to the product of the ambient atmospheric gas pressure and the gas volume after sampling into a tedlar bag.

Besides the O_2_:COD ratios (0%, 70%, 200%), the three other treatment variables applied were: Process temperature (200 °C and 300 °C); time (10 min and 60 min); and total feed solids (3.1% wt. and 7.7% wt.). In addition to the full factorial design, six additional experiments were produced at 140 °C and 240 °C and O_2_:COD ratios of 0%, 70% and 200% with feed material at 3.1% wt solids and processed for 60 min. Data from the factorial design and the additional experiments at 140 °C and 240 °C were included in the multivariate analyses described below.

In this study, we were interested in the impact of individual treatment variables on off-gas composition. However, in literature, one approach to understanding particularly non-oxidative HP processing of lignocellulosic materials has been to combine the temperature and time treatment variables into a severity factor log_10_ (R_0_). This factor expresses a kinetic dependence on temperature comparable to the Arrhenius equation (Overend et al., 1987; Ruiz et al., 2021). This approach has been attractive because it reduces the dimensionality of conditions for the analyses of results. However, by itself, the conventional severity factor has some significant limitations for application in this work. It assumes first order conversions during hydrothermal processing and that chemical reactions in the matrix have an activation energy typical for glycosidic bond cleavage of carbohydrates (14.75) (Ruiz et al., 2021). It also does not account for oxidative reactions and the factors which influence those. Nevertheless, for comparative purposes, the severity factors log_10_ (R_0_) for the treatments in this study were calculated using Eq. (1) (Overend et al., 1987; Ruiz et al., 2021):

(1) $$R_{0}=t\cdot exp^{\left[\frac{\left(T-100\right)}{14.75}\right]}$$

Where R_0_ is severity factor (min), t is time (min), T is reaction temperature (°C), 100 °C is base temperature, and 14.75 is constant related to the activation energy. The calculated severity factor log_10_ (R_0_) for the various treatments used in this study were: 3.0 at 140 °C, 60 min; 3.9 at 200 °C, 10 min; 4.7 at 200 °C, 60 min; 5.9 at 240 °C, 60 min; 6.9 at 300 °C, 10 min; and 7.7 at 300 °C, 60 min.

### 2.3 Gas analysis

The off-gas samples were analysed within 24 h for permanent gas components (CO_2_, H_2_, and CO) and VOC analyses. The permanent gas components were analysed using a gas chromatograph (Agilent 490 Micro GC) equipped with three channels with heated injectors at 50 °C; a Molecular sieve 5A (back-flush at 5.10 s, 90 °C, 150 kPa), a PoraPak Q column (no back-flush, 70 °C, 50 kPa) and a 5CB column (no back-flush, 80 °C, 150 kPa).

The VOC analyses were completed using gas chromatography mass spectrometry (GC-MS) and followed the internationally recognized method by VDA (2011). The analytical system consisted of a Multi-Purpose Sampler (Gerstel GmbH, Germany), operated in large volume injection-mode and equipped with a 2.5 mL syringe. A temperature controlled cooled injection system (CIS) (Gerstel GmbH) was used as an interface, cold trap and injection system for the subsequent GC-MS analysis. Samples and controls were analysed on an Agilent model 7890B/7000A GC/MS triple quadrupole instrument by automatic injection of a 10-mL sample from each bag onto Tenax adsorbent at -10 °C. Following sample injection onto the cold trap, the split was closed, and the CIS was ramped at 12 °C per sec to 230 °C for splitless transfer of the analytes to the capillary column. The helium carrier gas pressure was held constant at 18 psi. The GC was fitted with a 30 m × 0.250 mm i.d. fused silica capillary column, with a 1.4 μm coating (DB-624, Agilent J&W). The GC oven was programmed from an initial temperature of 35 °C (held for 2 min) followed by a 25 °C per min increase to 60 °C. The oven temperature was then increased by 20 °C per min to 220 °C (held for 2.5 min) and finally increased by 10 °C/min to 245 °C, and held for 10 min. Full scan mass spectra were acquired from 29 to 500 m/z. Electron impact ionization at 70 eV was employed, with an interface temperature of 230 °C and a source temperature of 250 °C. Full scan data were acquired and processed using Agilent MassHunter Workstation software (2016 version B.08.00).

Calibration curves were constructed using methyl methacrylate, toluene and o-xylene standards at concentrations 0, 0.1, 0.25, 0.5, and 1 ppm (MegaMix® Calibration mix standard; Restek, USA). Additional calibration curves were created using acetone (0, 1, 2.85, 5, 10 ppm) and furan (0, 1, 2.5, 5, 10 ppm) prepared immediately prior to analysis. These liquid standards were prepared in methanol and injected into 20 mL headspace vials. The standard vials were heated at 85 °C for 10 min whereby 1 mL was removed from the vial and injected onto the cold trap held at -10 °C then heated at 12 °C/min to 200 °C and subsequently loaded onto the column following the same chromatography conditions as the samples.

Identification of the analytes was accomplished by comparison of mass spectra and retention index data with literature, computer library data (NIST library 2012 NIST MS Search 2.0, and Scion's in-house Terpenes library), and comparisons with compound standards. Tentative identification was assigned where the library match was greater than 80%. On-column analyte concentrations were calculated from the calibration curves. When primary standards were not available, the closest standard based on structural similarity was used for quantitation and the response factors were assumed to be equal. Lighter VOCs (e.g. ethylene, methanol, etc) are not quantitatively retained on the Tenax cold-trap due to their lower heats of adsorption (Szulejko et al., 2013). Significant losses may have occurred for these compounds. However, losses were assumed equal for the various treatments investigated.

### 2.4 Statistical analyses

A linear model was used to test for the effects of experimental treatments on VOC production, both individual and groupings based on chemical similarities. Multivariate analysis of variance was used to test for the effects of experimental parameters on yields of individual compounds. All statistical tests were undertaken with the statistical program R (R development team, 2019).

The 69 individual VOC profiles per gas sample were examined using non-metric multidimensional scaling to show the ordination of the mass of the individual volatile organic compounds (μg VOC per g TS in feed) created during hydrothermal processing under various conditions. A Bray distance matrix was used to arrange data prior to ordination. Stress of 0.1 was considered a reasonable representation of all VOCs in two dimensions (Clarke, 1993). The Envfit function in the R library ‘vegan’ was used to fit environmental factors into the ordination (Oksanen et al., 2019).

## 3 Results and discussion

### 3.1 CO_2_, CO and H_2_ production

**Figure 1:**
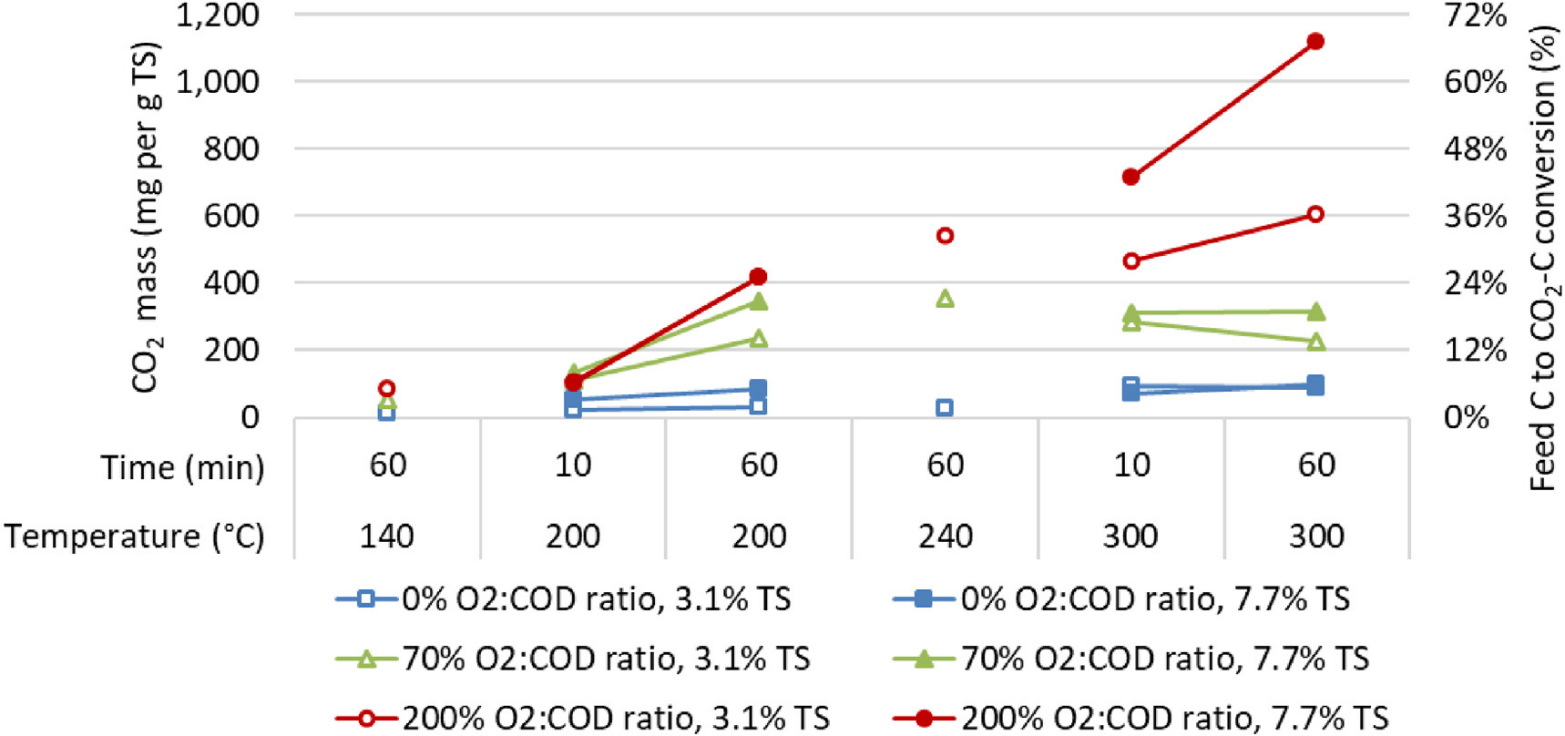
Production of CO_2_ (y-axis left) and feed carbon to CO_2_ carbon conversion efficiency (y-axis right) after hydrothermal processing at various temperatures, oxygen availability and processing times.

Increasing temperature, reaction time and oxidative environment increased the CO_2_ production and thus the degradation of feed organic carbon compounds (Figure 1), a trend also seen by others with cellulose and sewage treatment plant solids e.g.(Yousefifar et al., 2017a, 2017b). The production of CO_2_ was highest when sufficient oxygen was present at elevated temperatures of 300 °C and longer reaction times, where 35%–70% of the organic carbon feedstock was converted into CO_2_ (Figure 1) for low (3.1% TS) and high (7.7% TS) solids samples, respectively. At lower temperatures, with less O_2_ or shorter reaction times, much less organic carbon was converted, indicating that more VOCs and liquid phase reaction products like acetic acids were produced. This is consistent with the conclusions of Hübner et al. (2016), who found that only at 300 °C and above, VOCs ignite in the presence of O_2_ thereby converting to CO_2_.

**Figure 2:**
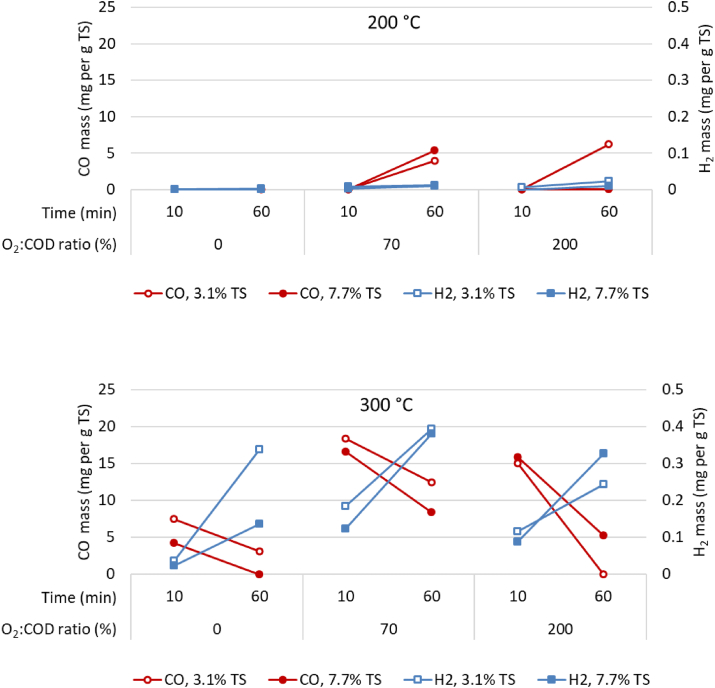
Carbon monoxide and hydrogen production after processing at 200 °C and 300 °C.

At 200 °C and 300 °C, the presence of oxygen in the headspace also led to significant CO production (Figure 2), although at 300 °C, CO decreased with reaction time. Hydrogen, on the other hand, was only observed at 300 °C, with production increasing with reaction times under both oxidative and non-oxidative conditions (Figure 2).

In our study, the greatest CO production occurred at 300 °C, at λ = 70% (Figure 2). Using the homogenised feedstock TS and feed C characteristics, the maximum conversion determined was then 1.7% of the feed carbon after 10 min, decreasing to a conversion of 1.2% of the feed carbon after 60 min. Formation and oxidation of CO in hydrothermal processing has also been observed in other studies, such as Hübner et al. (2016) whereby the oxidation rate of CO was considerably influenced by the reaction temperature. At temperatures of 360 and 470 °C, around 8% of the TOC was first converted to CO and consecutively oxidized to CO_2_ (Hübner et al., 2016). They postulated that formation of CO from organic carbon occurred in an independent reaction parallel to the formation of CO_2_ (with or without oxygen involvement), as opposed to all organic carbon being converted to CO before further oxidation to CO_2_. This concurs with the results we observed in this experiment. Possible mechanisms for CO production could be, for example, via acetaldehyde, formic acid (Hübner et al., 2016) and/or methanol (Mishra et al., 1995), all of which have been measured in liquors from subcritical wet oxidation.

In general, conversion of CO to CO_2_ can occur by oxidation in the presence of molecular oxygen, or by a reaction with water (water gas shift with H_2_ as co-product) at both comparatively low (210 °C–330 °C) and high (>350 °C) temperatures (Holladay et al., 2009). Although low temperature water gas shift reactions typically occur in the presence of an inorganic catalyst, the presence of heavy metals such as copper (Rose et al., 2015), or other inorganics (Krueger et al., 2020) in faeces feedstock make it conceivable that catalysts were present and low temperature water gas shift reactions occurred. The low temperature range (210 °C–330 °C) at which the water gas shift reaction can occur matches the temperature (300 °C) at which hydrogen formation was observed in this study. Under non-oxidative conditions at 300 °C, the conversion of CO into CO_2_ would be expected to occur via the water gas shift reaction because the alternatives require oxygen. This concurred with our study when at a temperature of 300 °C, the simultaneous decrease of CO and increase of H_2_ potentially indicated the occurrence of water gas shift reaction. At 3.1% TS feedstock, such conversion was (on a molar basis) more predominant when no oxygen was present than when oxygen was present. At 0% O_2_:COD ratio, a 1 mol CO decrease coincided with 0.97 mol H_2_ increase while at 70% O_2_:COD ratio, a 1 mol CO decrease coincided with 0.5 mol H_2_ increase, and at 200% O_2_:COD ratio, a 1 mol CO decrease coincided with only 0.12 mol H_2_ increase. This suggested that when oxygen is present, there is competition between water gas shift and oxygen oxidation reactions.

### 3.2 VOC production

A total of 69 VOCs were quantified (Table 1). These compounds were grouped by chemical functionality from a range of chemical classes: aldehydes, ketones, alcohols, furans, pyrroles, sulphur compounds, nitriles, aromatics, and hydrocarbons. Half of those quantified compounds contained oxygen, such as in aldehydes, ketones, and alcohols, 7% contained nitrogen and 9% contained sulphur. The three most prevalent individual compounds were furan, acetone and 1-methyl-cyclopentene. There were 15 VOC compounds that were found in all off-gas samples (Table 1). Rao et al. (2018) presented the top 10 VOCs measured from hydrothermal liquefaction of the anaerobic digestate from food and dairy farm waste (300 °C/λ = 0%, 60 min), of which 8 were also identified here, the most abundant being butanone and acetone.

**Table 1:** Number of VOCs identified per chemical grouping, the VOCs identified in every treatment, and significant difference levels ∗∗P < 0.01, ∗P < 0.05, and ns = not significant (P > 0.05) for total volatile organic carbon and chemical classes of compounds mass in the headspace after processing under the various conditions.

Chemical grouping	Total	Compounds identified in every treatment	O_2_:CODratio	Temp	Total solids	Time
Alcohol	4	cyclobutanol	∗	∗∗	ns	ns
Aldehyde	12	butanal; 3-methylbutanal; 2-methylbutanal; pentanal; hexanal				
Ketone	11	acetone; 2-butanone; 3-methyl-2-butanone; 2-hexanone				
Nitrile	1	none				
Aromatic	6	toluene	ns	∗∗	ns	ns
Hydrocarbon^1^	18	4-methyl-1,4-hexadiene	ns	∗∗	ns	ns
Furan	8	furan; 2-methylfuran; 2-ethylfuran	ns	ns	ns	ns
Pyrrole	3	none				
Sulphur compound	6	none	∗∗	ns	ns	ns
Total VOC	69	15	∗∗	∗∗	ns	∗∗

1 Contained alkenes and one alkane.

The factors that most influenced total VOC mass production were in order: temperature > O_2_: COD ratio λ > time. Altering the total solids content did not significantly impact on the yield of the different chemical classes and total VOC (Table 1).

The abundance of different VOC classes (measured as VOC mass per unit mass of input feed) are provided as a heatmap and were lowest at the low temperature of 140 °C, where hydrothermal non-oxidative and wet oxidation reactions would be very slow (Table 2). Temperature had a significant effect (P < 0.05) on yields of aldehydes, ketones, alcohols, nitriles, hydrocarbons and aromatics but not on yields of sulphur compounds, furans and pyrroles, while the oxygen ratio (λ) influenced the aldehydes, ketones, alcohols, nitriles, and sulphur compound yields (Table 1). The VOC abundances typically increased with temperature when the O_2_:COD ratio was limited at either λ = 0% or 70%. Conversely, when the oxygen levels were not limiting (λ = 200%), the VOC abundances increased until 200 °C then decreased with higher temperatures. At each temperature except 200 °C, VOC abundances peaked at λ = 70%. This indicated that an insufficient oxygen supply relative to the organic matter present (measured as COD) provided conditions that were amenable to net VOC generation. The best observed conditions for minimising total VOC production were either at a very low temperature of 140 °C, or at high temperatures (300 °C) with an oversupply of oxygen (λ = 200%). Low VOC generation at 140 °C would suggest that this temperature is too low for significant hydrothermal conversion of our substrate. At 300 °C and an oversupply of oxygen, the low VOC generation indicates that these conditions would be optimal for minimising VOC generation during effective hydrothermal processing of our substrate. This finding was supportive of our hypothesis.

**Table 2:**
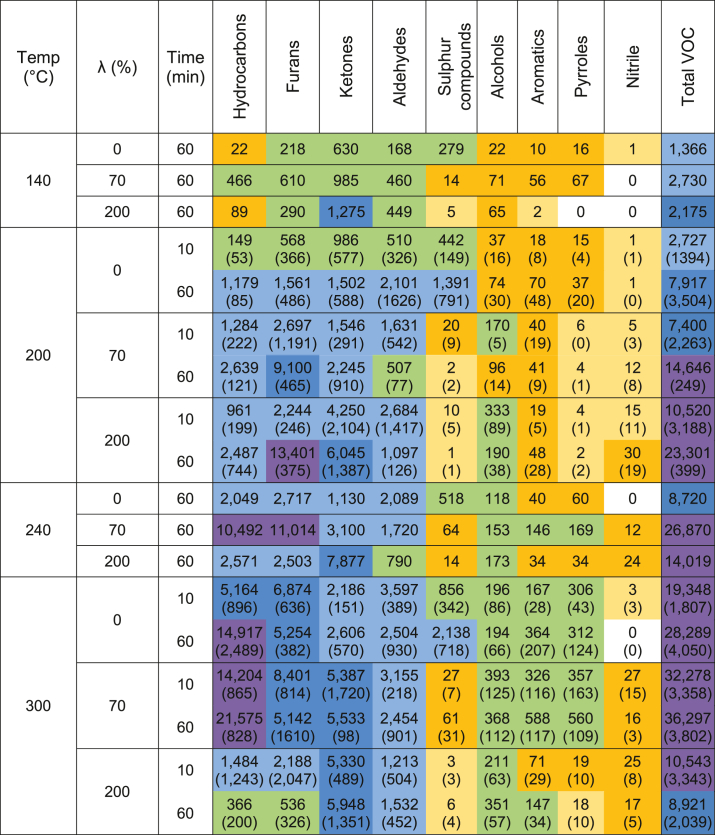
The production of volatile organic carbons (nmol/g TS) in the various chemical classes for each of the treatment combinations and the average summed total VOC. Values represent an average of the 3.1% and 7.7%TS results when both available because the TS factor was found to be not significant. Values in brackets indicating the Standard Error (SE). Colours indicate scale range: 0 nmol/g TS, 0–10 nmol/g TS, 10–100 nmol/g TS, 100–1,000 nmol/g TS, 1,000–5,000 nmol/g TS, 5,000–10,000 nmol/g TS, 10,000–50,000 nmol/g TS..

**Figure 3:**
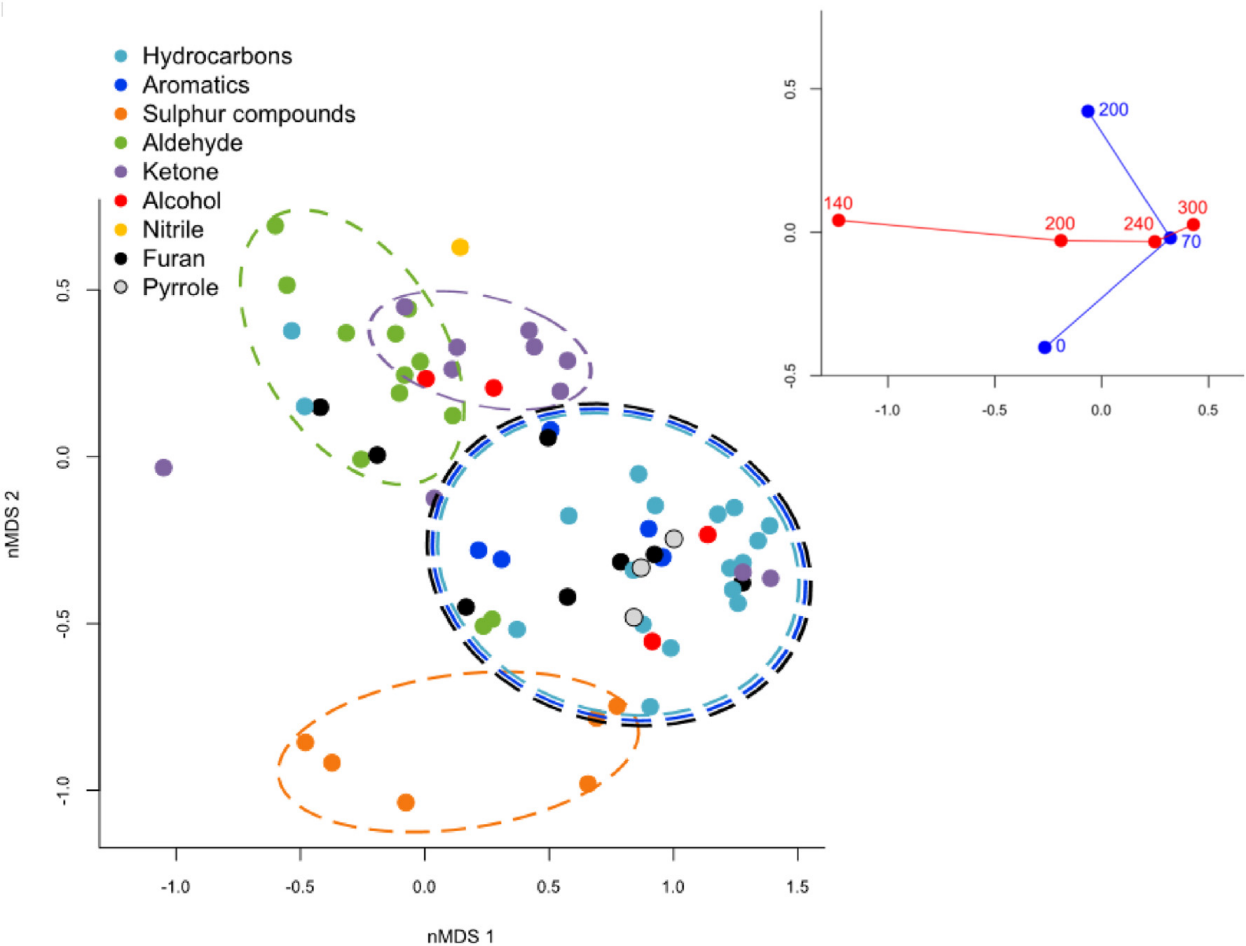
Non-metric multidimensional scaling diagram of the mass of 69 individual volatile organic compounds (μg VOC per g TS) created during hydrothermal processing under various conditions. The statistically significant experimental factors Temperature (°C) () and O_2_:COD ratio (λ, %) () were related to the composition of VOCs and are represented on the inset plot.

A non-metric multidimensional scaling (nMDS) analysis was completed and confirmed a strong influence of temperature and oxidative state (λ). Their influence on the production of each of the identified VOCs was indicated by location in the vertical (λ) or horizontal (temperature) planes as indicated by the plot inset in Figure 3. The sulphur containing VOCs, for example, were mainly produced under non-oxidative conditions at low to medium temperatures while aldehydes made up a large fraction of the compounds produced under oxidative conditions at low temperatures.

**Figure 4:**
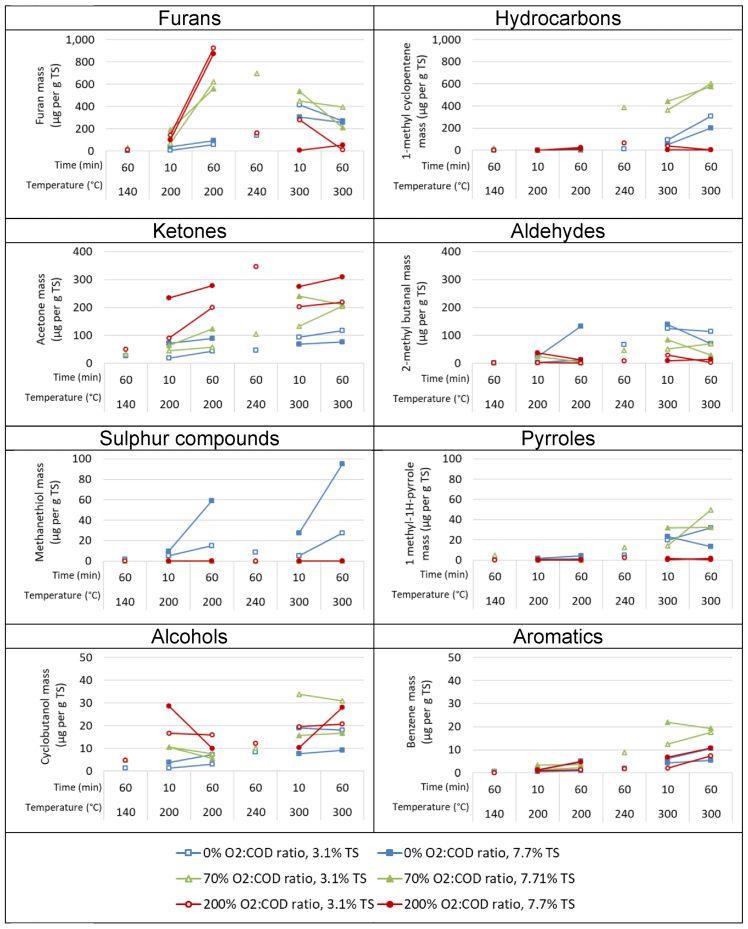
The most abundant compound (shown on y-axis) within each class as mass produced per mass feed after hydrothermal processing at various temperatures, oxygen supply levels (O_2_:COD, λ), processing times and total feed solids contents.

To compare individual compounds in more detail and the impact that oxygen supply had on them, the production of the most abundant VOC within each compound class was shown under the various processing conditions (Figure 4). The most abundant compound overall was furan. Jin et al. (2005) reported that furans are intermediary products during thermal hydrolysis processing (temperature range 250 °C–350 °C) of carbohydrate biomass, and in the presence of oxygen were subsequently converted to acetic acid during wet oxidation. Furan in our study was most prevalent at 200 °C in the presence of oxygen, with yields increasing when oxidation time was prolonged at this temperature. Increasing the temperature to 300 °C increased furan concentrations at non-oxidative conditions but decreased furan concentrations markedly in the presence of excess oxygen. Furans are directly derived via thermal decomposition from polysaccharides such as the hemicelluloses xylan and glucomannan, and also from cellulose (Borén et al., 2018; Werner et al., 2014). It was suggested that furans were generated during steam explosion at treatment temperatures greater than 200 °C. In an interesting exploration of non-oxidative hydrothermal processing, Usman et al. (2019) describes furan degradation occurring at higher temperatures of 240 °C–300 °C during the treatment of lignocellulosic biomass. In our study, after 60 min with an oversupply of oxygen, lower concentrations of furans were found at 300 °C than at 200 °C.

The initial feedstock composition will play a factor in the nature and abundance of created VOCs. The impact of feedstock composition was reported by (Molina-Garcia et al., 2017) who indicated that VOC composition after deep-frying a range of oils was highly associated with the initial composition. Our feedstock consisted of faeces, urine and toilet paper (mainly cellulose) whereby the faeces composition was dependent on dietary intake but typically contains 25%–54% bacterial biomass, 2%–25% protein, 25% carbohydrate and 2%–15% undigested lipids (Rose et al., 2015).

### 3.3 Effect of temperature

The formation of total VOCs, and specifically aromatics, hydrocarbons, aldehydes, ketones, alcohols, and furans (Table 1), significantly increased with increasing temperatures especially under limited or no oxygen conditions. Under such hydrothermal processing conditions without oxygen, Usman et al. (2019) summarised the fate of lipids, proteins, carbohydrates and lignin with increasing processing temperatures. At about 140 °C–240 °C, lipids are converted into fatty acids and glycerol, which at 265 °C–300 °C further react into aldehydes. Carbohydrates (like cellulose and hemicellulose) hydrolyse to monosaccharides at 120 °C–200 °C. These carbohydrates can react further at 220 °C–300 °C to form ketones, alcohols and furfurals. Proteins are hydrolysed to amino acids at 85 °C–150 °C. In turn, amino acids convert to ammonia and organic acids at 185 °C–250 °C, or they can convert at about 200 °C–245 °C in the presence of monosaccharides, into melanoidins via the Maillard reaction, best known for browning of food. Lignin only converts to phenols at temperatures above 240 °C. Further conversion into nitrogen and oxygenic heterocyclic compounds started for melanoidines, and ketones at around 270 °C–280 °C and for furfurals at 230 °C and above (Usman et al., 2019). This would explain why in our study there was much more furfural at 300 °C than at 200 °C (data not shown) and the influence of temperature increase on the formation of aromatics, hydrocarbons, aldehydes, ketones, alcohols (Figure 1), particularly when oxygen is absent or supply is limited.

### 3.4 Effect of oxygen

In our study, less total VOCs, and specifically hydrocarbons, aldehydes, sulphur compounds (Table 1), and furans (Figure 4) were formed when temperatures increased to 300 °C, in the presence of sufficient oxygen. In addition to the chemical reactions occurring during non-oxidative hydrothermal processing such as hydrolysis, thermal decomposition, dehydration, decarbonylation and decarboxylation (Usman et al., 2019), the presence of oxygen generates highly reactive oxidative radical species (Baroutian et al., 2016; Debellefontaine and Foussard, 2000). At conditions below the critical point of water (T < 374 °C and p < 22.1 MPa), most oxidation reactions occur in the liquid phase among dissolved compounds (Hübner et al., 2016). Oxygen must diffuse from the gas phase into the liquid phase in order to oxidise dissolved organic material, implicating that under limited mass transfer rates of oxygen, the feed solids concentration can affect VOC composition (Hübner et al., 2016). In our study, there was no significant difference between low and high solids yields, suggesting that oxygen mass transfer rates were not limiting.

For a less complicated dry oxidation system (Everaert and Baeyens, 2004), the thermal combustion of VOCs such as hydrocarbons were independent of oxygen concentrations and compounds with the strongest bonds created the most stable intermediate compounds and highest activation energies. When temperatures are above 250 °C, oxygen solubility in liquid increases beyond that at room temperature (Debellefontaine and Foussard, 2000), thus increasing the oxygen available for oxidation reactions in liquids. This higher oxygen availability in a wet environment could explain why, in our study, organic compounds at 300 °C were more effectively oxidised than at 200 °C, and thus resulted in lower VOCs in the off-gas.

### 3.5 Effect of time

Increasing reaction times from 10 min to 60 min, mostly increased total VOC yields, increased CO_2_ yields and decreased CO yields in this study. The fact that time did not have a significant impact on many VOCs classes produced, indicated that the effect of time on individual VOC classes was small and only showed for total VOCs when all VOCs were considered together (Table 1). Alternatively, a large proportion of the VOCs could already have been oxidised within the first 10 min at temperatures ranging from 220 °C to 290 °C (Li et al. (1991), therefore not showing differences between 10 min and 60 min. Nevertheless, reaction times could vary considerably depending on the activation energy for a specific chemical reaction, the specific composition of the feed stock, and the presence of oxygen.

Hübner et al. (2016) reported that hydrothermal oxidation reactions of faecal sludge at 470 °C converted all TOC and CO to CO_2_ in about 25 min while at 360 °C and 25 min, the TOC was converted, but 6% of carbon was still recovered as CO. In this study, complete conversion to CO_2_ was unlikely due to the lower temperature range of 140 °C–300 °C. Indeed, Figure 1 indicated that conversions to CO_2_ at 10 min ranged from 1.2% to 42% with a median of 6.5% across all treatments. At 60 min, the conversion percentage to CO_2_ ranged from 0.8% to 66% with a median of 14%.

The decrease of CO and the matching increase in CO_2_ and H_2_ did appear to occur within the 10–60 min time frame at 300 °C. Indicating that, if minimising CO production was the main focus, the best conditions in this study were processing at 300 °C for 60 min under either non-oxidative conditions (0% O_2_:COD) or in the presence of plenty of oxygen (200% O_2_:COD). However, at 0% O_2_:COD, the total VOC would be 2 to 3 times greater than at 200% O_2_:COD.

### 3.6 Implications

Based on a worst-case scenario of an average number of 6 people per household (United Nations, 2017) and an average daily TS production of 39 g dry faeces per person (Rose et al., 2015), 234 g TS would need to be processed per household per day. In this study, each run processed either 0.155 g TS or 0.38 g TS in 10 or 60 min. This would indicate that to process toilet waste from a 6-person household on a daily basis several reactors would need to run concurrently or the reactor volume would need to be scaled up taking account of the increased safety risk associated with bigger volume pressure vessels.

Exposure to VOCs can be a health concern. Dehydration of carbohydrates during thermal hydrolysis conditions forms furans (Jin et al., 2005) while sulphur compounds likely originate from the degradation of sulphur containing amino acids in the proteins. Unsaturated fatty acids during processing at different temperatures turned into VOCs like furans, alkanals, benzene, toluene (Zhang et al., 2019), alkanes and alkenes (Juita et al., 2012), all of which were also detected in our study. The amino acids and sugars formed during sub-critical hydrothermal processing formed nitrogen heterocyclic compounds like pyrroles (Usman et al., 2019). Recurring VOCs of concern that were present in this study included aldehydes (acetaldehyde, pentanal, hexanal, heptanal), acroleins, benzene, 1,3 dimethyl benzene, toluene, m xylene and dimethyl disulphide. Aromatics like benzene, toluene, ethylbenzene are known carcinogens while some aldehydes contribute to a range of mutagenic and cytotoxic mechanisms (O'Brien et al., 2005).

An hourly indoor emission threshold for VOC was given as 187 ppbv in ISO 30500 (2018). The reactor headspace contained VOC concentrations between 22 ppmv and 1189 ppmv for the various processing conditions. After 10 min, this equated to a median of 138 ppmv VOCs and a median gas volume of 381 mL, and after 60 min this equated to a median of 251 ppmv VOCs and a median gas volume of 277 mL. These median indoor emissions would require a 738 times dilution after 10 min and 1342 times dilution after 60 min. This indicated that venting the reactor headspace outside and scrubbing for gaseous emissions is desirable.

In this study, the treatment conditions to best eliminate sulphur containing VOCs involved oxidative hydrothermal processing at any temperature (140 °C–300 °C) in the presence of 200% O_2_:COD ratio (Table 2). Benzene and toluene were mostly formed at 300 °C at 0% and 70% O_2_:COD ratio. Aldehydes were lowest at 300 °C and 200% O_2_:COD. And although optimal conditions for individual VOCs are likely to be different for many of them, at 300 °C in the presence of an oversupply of oxygen (200% O2:COD), the lowest yields of VOCs were present in the off-gas of our hydrothermal treatment process. Assuming outdoor emissions into ambient air, and based on hourly ambient air emission thresholds for VOC of 12 ppmv and CO of 80 ppmv (Tüv Süd Asia Pacific Pte. Ltd., 2016), processing at 300 °C and 200% O_2_:COD for 60 min would still require a 6 to 12 times dilution for the VOC emissions of 72–143 ppmv to meet the threshold for VOC emissions and up to 23 times dilution for the CO emissions of up to 1800 ppmv to meet the threshold for CO emissions.

Therefore, the off-gas may need to undergo some additional treatment, for example, an exhaust scrubber would enable one to minimise VOC emissions and recycle the scrubber effluent by hydrothermal processing. Irrespectively of the management of the VOC emissions, it has become clear that the development of the hydrothermal processing of toilet waste needs to consider VOC emissions as a critical part of the technology development.

## 4 Conclusion

To minimise VOC emissions during effective hydrothermal processing of toilet waste, the most important conditions were elevated temperatures and oversupply of oxygen compared to feed organic content. When those conditions were applied for prolonged time periods, CO concentrations in the off gas were also minimised. Low formation of VOC and CO compounds during hydrothermal processing is desirable to lower potential impacts on the receiving environment, and to minimise potential health effects. Finally, awareness of VOCs and CO emissions will be essential to determine what type of exhaust system will be required for safe hydrothermal processing of toilet waste.

E-supplementary data of this work can be found in the online version of the paper.

## Declarations

### Author contribution statement

Gerty J.H.P. Gielen & John P. Andrews: Conceived and designed the experiments; Performed the experiments; Analyzed and interpreted the data; Contributed reagents, materials, analysis tools or data; Wrote the paper.

Christine M. Karbiwnyk: Performed the experiments; Analyzed and interpreted the data; Contributed reagents, materials, analysis tools or data.

Mark J.C. Riddell: Conceived and designed the experiments.

Sean W. Husheer: Analyzed and interpreted the data; Contributed reagents, materials, analysis tools or data.

Daniel J. Gapes: Conceived and designed the experiments; Wrote the paper.

### Funding statement

Daniel J. Gapes was supported by Bill and Melinda Gates Foundation [OPP1155615].

### Data availability statement

Data included in article/supplementary material/referenced in article.

### Declaration of interests statement

The authors declare no conflict of interest.

### Additional information

No additional information is available for this paper.
