# Supplementary material for: Hydrothermal conversion of toilet waste: effect of processing conditions on gas phase emissions
Source: Heliyon. 2022 Jun 13;8(6):e09708. doi: 10.1016/j.heliyon.2022.e09708 (PMC9213707; doi:10.1016/j.heliyon.2022.e09708)
Supplement: Supplementary data [file mmc1.docx]

## Supplementary data for Manuscript titled:

Hydrothermal conversion of toilet waste: effect of processing conditions on gas phase emissions

**Table S1. Detected compounds retention time and calibration parameters**

| **Compound** | **Retention time** | **Calibration standard** | **Regression slope** |
| --- | --- | --- | --- |
| **Propene** | 2.26 | **acetone** | **5,000,000** |
| **2-Butene** | 2.67 | **acetone** | **5,000,000** |
| **cyclobutanol** | 2.83 | **furan** | **8,000,000** |
| **acetaldehyde** | 2.83 | **acetone** | **5,000,000** |
| **Methanethiol** | 2.93 | **acetone** | **5,000,000** |
| **Pentane** | 3.51 | **acetone** | **5,000,000** |
| **Furan** | 3.76 | **furan** | **8,000,000** |
| **2-Pentene** | 3.82 | **acetone** | **5,000,000** |
| **Acetone** | 3.97 | **acetone** | **5,000,000** |
| **1,3-Cyclopentadiene** | 4.20 | **acetone** | **5,000,000** |
| **Cyclopentene** | 4.31 | **acetone** | **5,000,000** |
| **Propanal, 2-methyl-** | 4.70 | **acetone** | **5,000,000** |
| **Methacrolein** | 4.86 | **methyl methacrylate** | **7,000,000** |
| **2-Pentene, 2-methyl-** | 4.93 | **acetone** | **5,000,000** |
| **Furan, 2-methyl-** | 5.09 | **furan** | **8,000,000** |
| **Butanal** | 5.18 | **acetone** | **5,000,000** |
| **Furan, 3-methyl-** | 5.24 | **furan** | **8,000,000** |
| **2-Butanone** | 5.31 | **acetone** | **5,000,000** |
| **1-Hexene, 3-methyl-** | 5.55 | **acetone** | **5,000,000** |
| **Cyclopentene, 1-methyl-** | 5.63 | **acetone** | **5,000,000** |
| **(Z),(Z)-2,4-Hexadiene** | 5.87 | **acetone** | **5,000,000** |
| **isobutynitrile** | 5.93 | **furan** | **8,000,000** |
| **Benzene** | 5.99 | **o-xylene** | **10,000,000** |
| **Butanal, 3-methyl-** | 6.05 | **acetone** | **5,000,000** |
| **Butanal, 2-methyl-** | 6.16 | **acetone** | **5,000,000** |
| **2-Butenal, 2-methyl-, (E)-** | 6.18 | **acetone** | **5,000,000** |
| **1,4-Hexadiene, 4-methyl-** | 6.39 | **acetone** | **5,000,000** |
| **Furan, 2-ethyl-** | 6.39 | **furan** | **8,000,000** |
| **3,5-Dimethylcyclopentene** | 6.43 | **acetone** | **5,000,000** |
| **2-Butanone, 3-methyl-** | 6.53 | **acetone** | **5,000,000** |
| **Pentanal** | 6.61 | **acetone** | **5,000,000** |
| **3-Pentanone** | 6.62 | **acetone** | **5,000,000** |
| **Furfural** | 6.62 | **furan** | **8,000,000** |
| **Furan, 2-ethyl-5-methyl-** | 6.88 | **furan** | **8,000,000** |
| **1-Methylcyclohexa-1,3-diene** | 6.93 | **methyl methacrylate** | **7,000,000** |
| **Cyclobutane, (1-methylethylidene)-** | 6.97 | **methyl methacrylate** | **7,000,000** |
| **Cyclopentene, 3-ethyl-** | 7.02 | **acetone** | **5,000,000** |
| **2-Hexene, 4-methyl-, (E)-** | 7.05 | **acetone** | **5,000,000** |
| **3-Methoxycyclohexene** | 7.10 | **o-xylene** | **10,000,000** |
| **Disulfide, dimethyl** | 7.22 | **methyl methacrylate** | **7,000,000** |
| **1H-Pyrrole, 1-methyl-** | 7.25 | **furan** | **8,000,000** |
| **3-Pentanone, 2-methyl-** | 7.31 | **toluene** | **20,000,000** |
| **Hex-4-yn-3-one** | 7.35 | **toluene** | **20,000,000** |
| **Toluene** | 7.39 | **toluene** | **20,000,000** |
| **2-Imidazolidinone** | 7.41 | **toluene** | **20,000,000** |
| **2-Pentenal, 2-methyl-** | 7.46 | **toluene** | **20,000,000** |
| **Thiophene, 3-methyl-** | 7.50 | **methyl methacrylate** | **7,000,000** |
| **2-Octen-1-ol, (E)-** | 7.55 | **acetone** | **5,000,000** |
| **Thiophene, 2-methyl-** | 7.62 | **methyl methacrylate** | **7,000,000** |
| **3-Heptyne, 5-methyl-** | 7.68 | **methyl methacrylate** | **7,000,000** |
| **Furan, 2,3,5-trimethyl-** | 7.84 | **furan** | **8,000,000** |
| **2-Hexanone** | 7.85 | **acetone** | **5,000,000** |
| **Hexanal** | 7.93 | **acetone** | **5,000,000** |
| **1-Ethyl-5-methylcyclopentene** | 8.03 | **furan** | **8,000,000** |
| **3,5-Heptadien-2-ol, 2,6-dimethyl-** | 8.12 | **acetone** | **5,000,000** |
| **1H-Pyrrole, 2-ethyl-** | 8.17 | **furan** | **8,000,000** |
| **Methyl ethyl disulfide** | 8.38 | **methyl methacrylate** | **7,000,000** |
| **m-xylene** | 8.59 | **o-xylene** | **10,000,000** |
| **Benzene, 1,3-dimethyl-** | 8.67 | **o-xylene** | **10,000,000** |
| **o-Xylene** | 8.68 | **o-xylene** | **10,000,000** |
| **2-Butylacrolein** | 8.68 | **methyl methacrylate** | **7,000,000** |
| **4-Heptanone** | 8.79 | **acetone** | **5,000,000** |
| **1H-Pyrrole-2-ethanamine, 1-methyl-** | 8.84 | **furan** | **8,000,000** |
| **Styrene** | 9.02 | **toluene** | **20,000,000** |
| **2-Heptanone** | 9.04 | **acetone** | **5,000,000** |
| **Heptanal** | 9.14 | **acetone** | **5,000,000** |
| **Disulfide, methyl propyl** | 9.53 | **methyl methacrylate** | **7,000,000** |
| **Furan, 2-pentyl-** | 9.89 | **furan** | **8,000,000** |
| **Acetophenone, 4'-hydroxy-** | 9.90 | **acetone** | **5,000,000** |


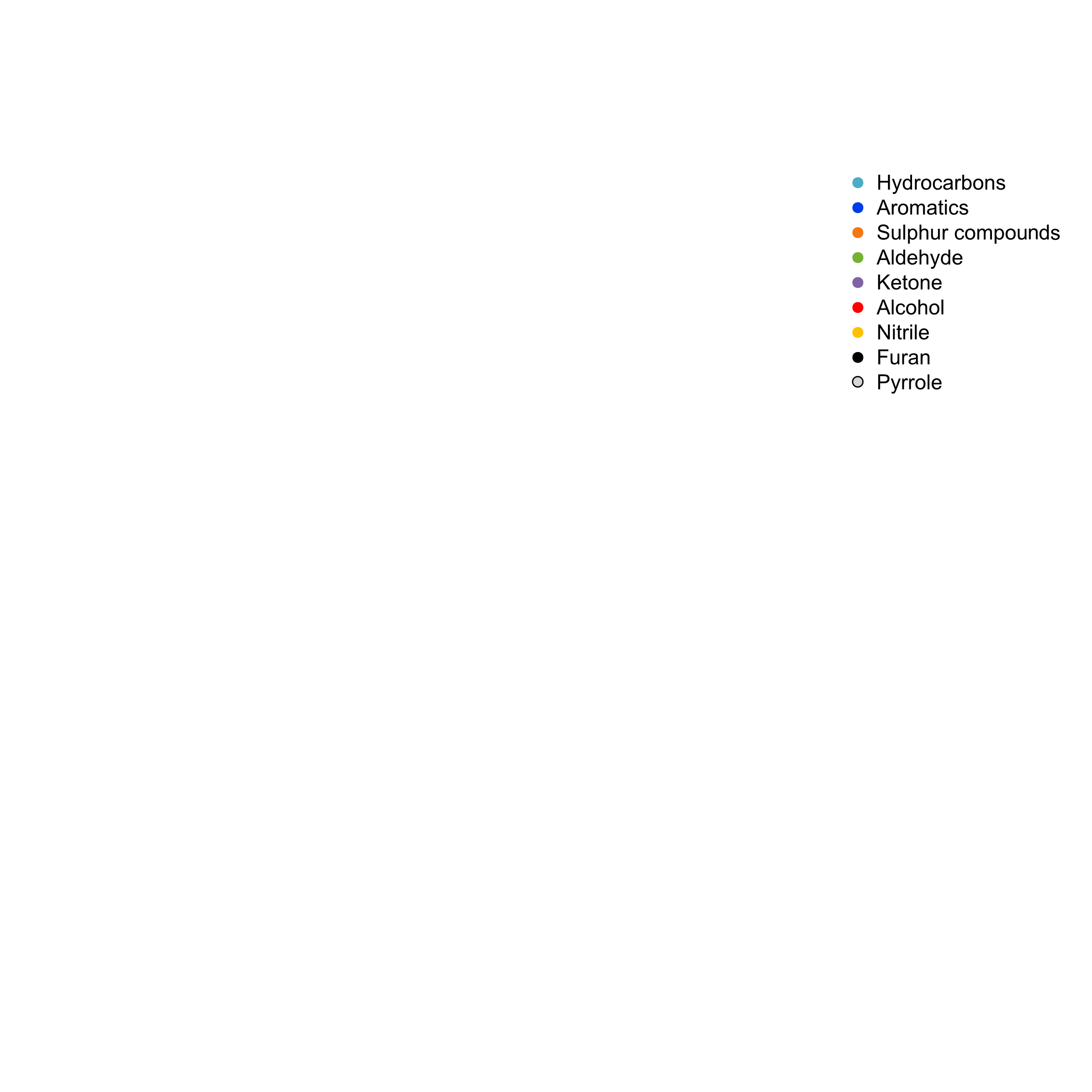
Fig. S1. Non-metric multidimensional scaling diagram of the mass of 69 individual volatile organic compounds (µg VOC per g TS) created during hydrothermal processing under various conditions. The statistically significant experimental factors: Temperature (
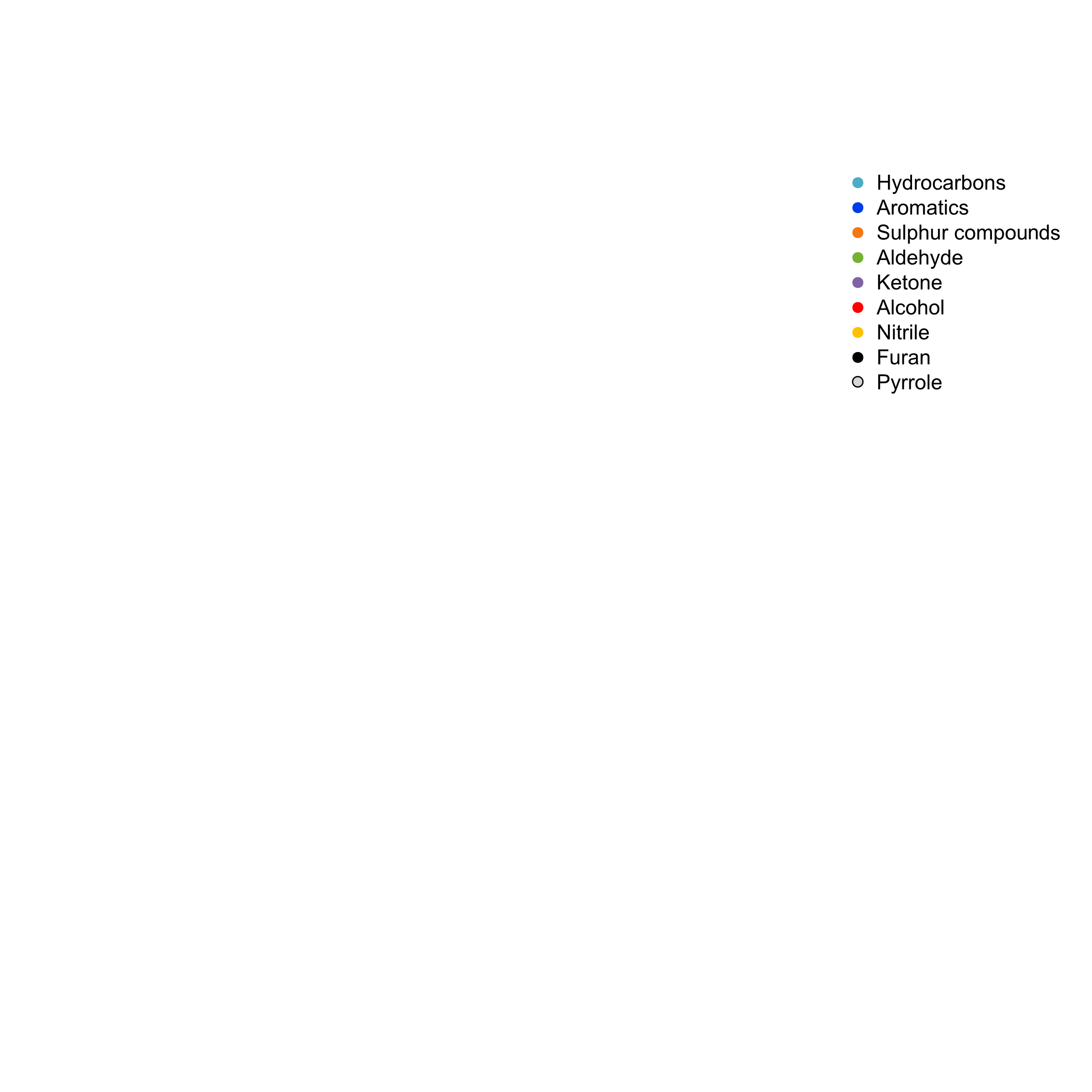
 ) and O_2_:COD ratio ( ) factors were related to the composition of VOCs.


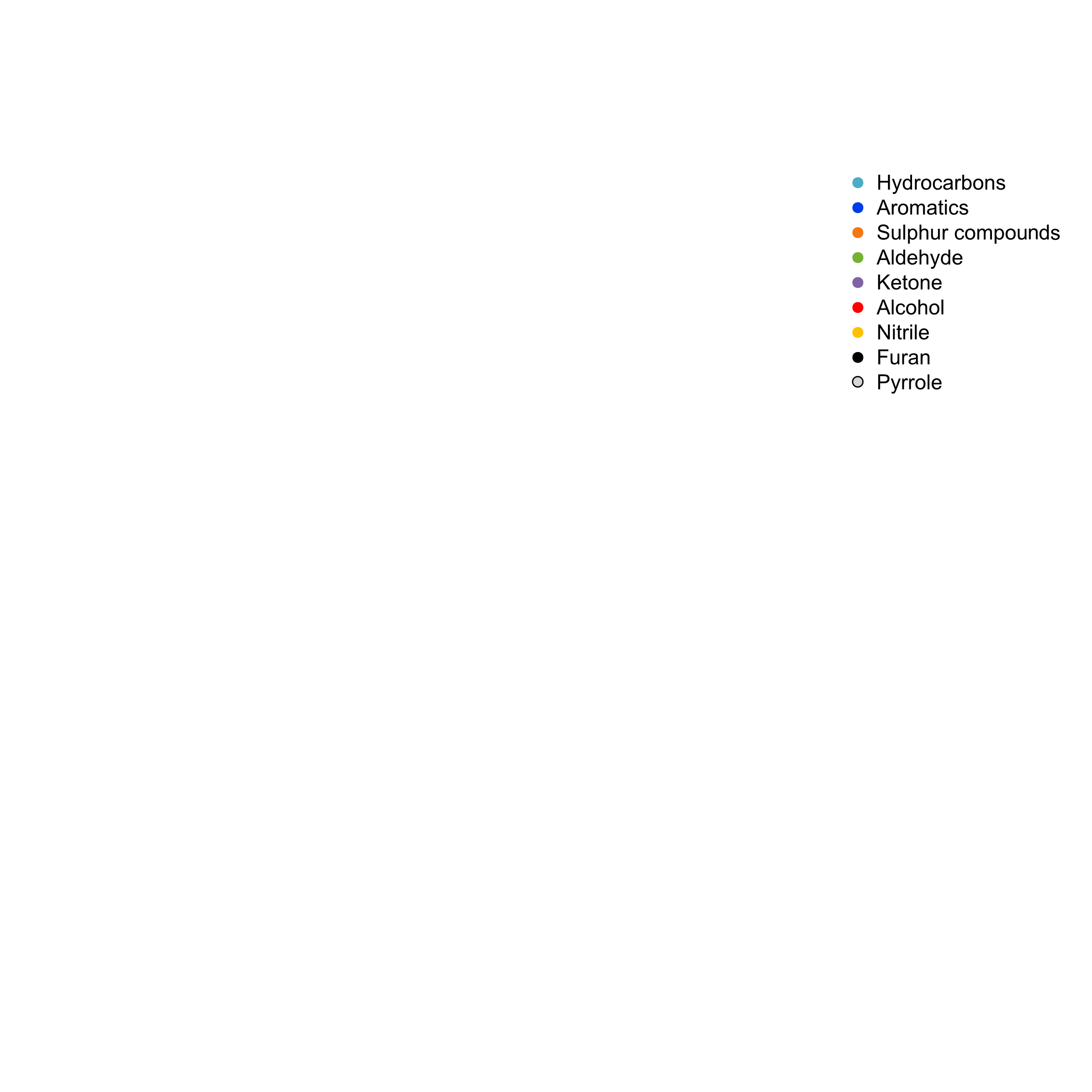

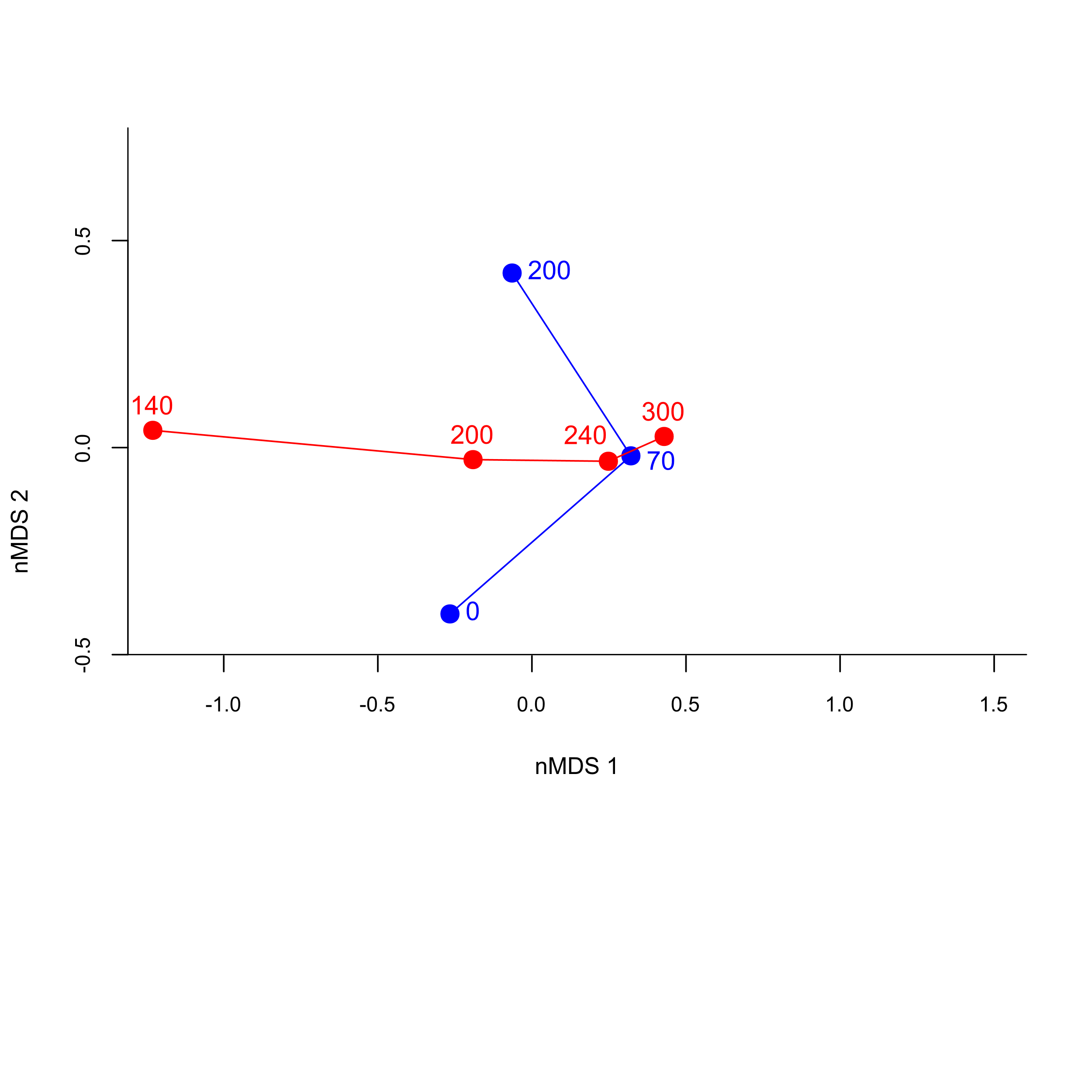

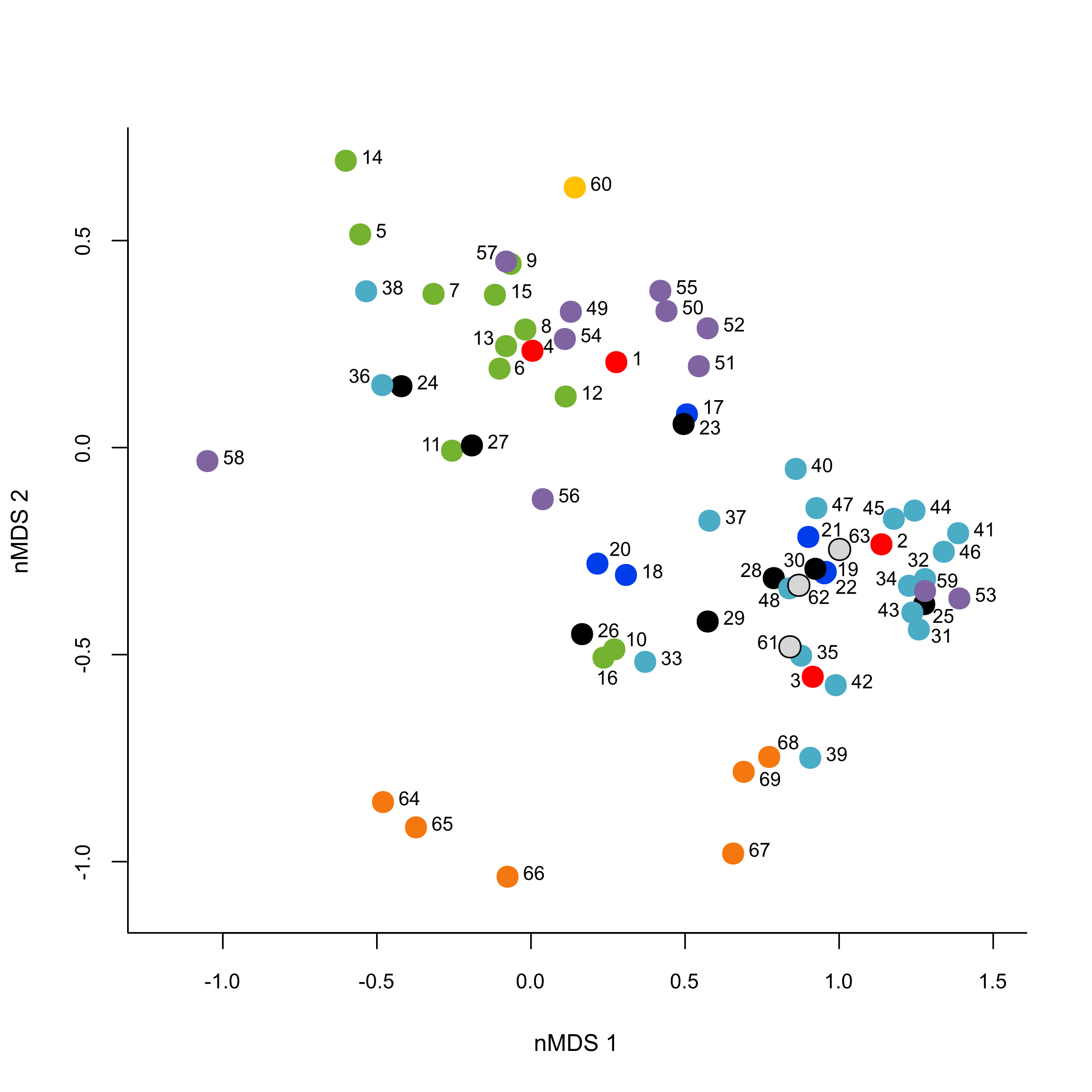


Alcohol **1**: 2-Octen-1-ol, (E); **2**: 3-Methoxycyclohexene; **3**: 3,5-Heptadien-2-ol, 2,6-dimethyl; **4**: Cyclobutanol


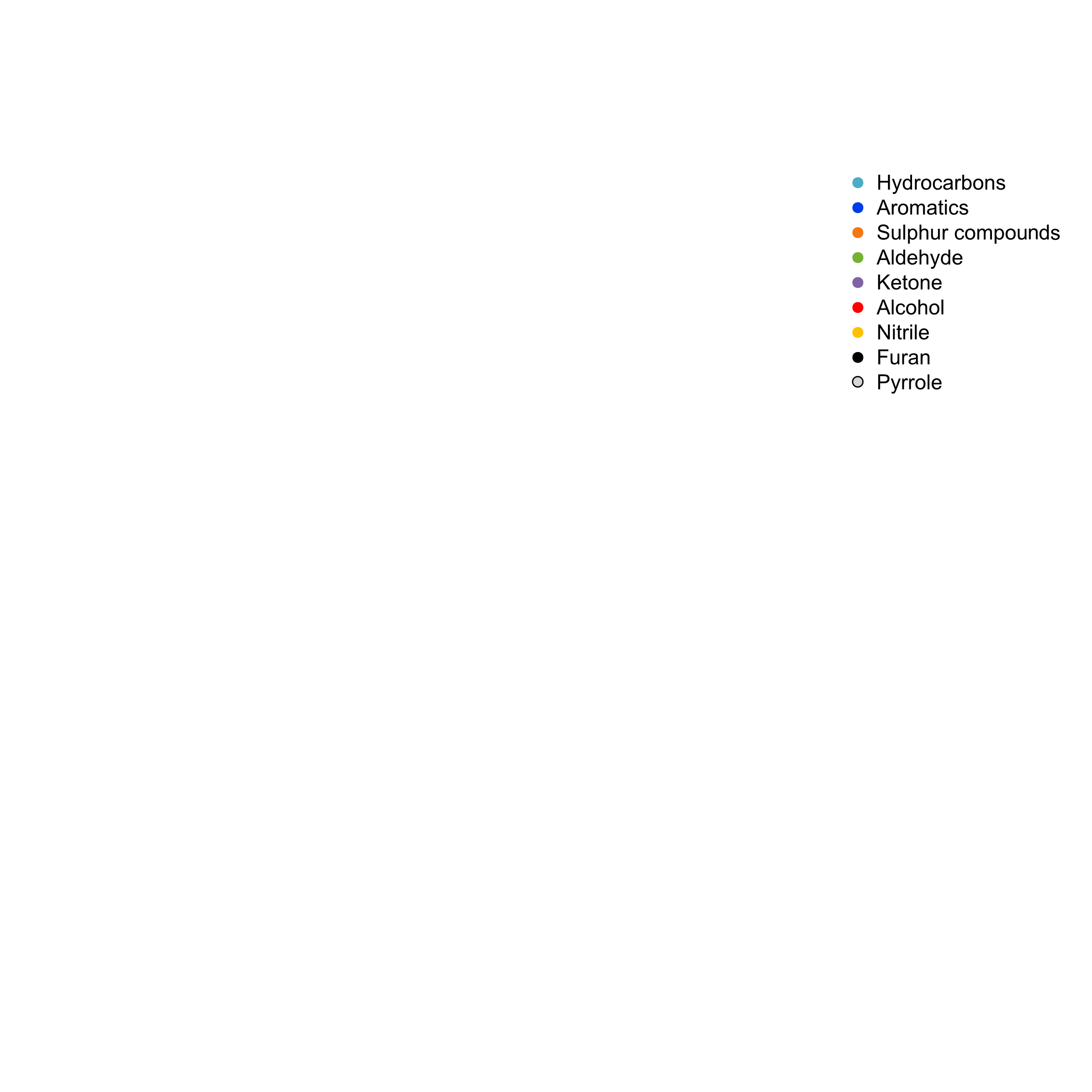
 Aldehyde **5**: 2-Butenal, 2-methyl-, (E); **6**: 2-Butylacrolein; **7**: 2-Pentenal, 2-methyl; **8**: Acetaldehyde; **9**: Butanal; **10**: Butanal, 2-methyl; **11**: Butanal, 3-methyl; **12**: Heptanal; **13**: Hexanal; **14**: Methacrolein; **15**: Pentanal; **16**: Propanal, 2-methyl;


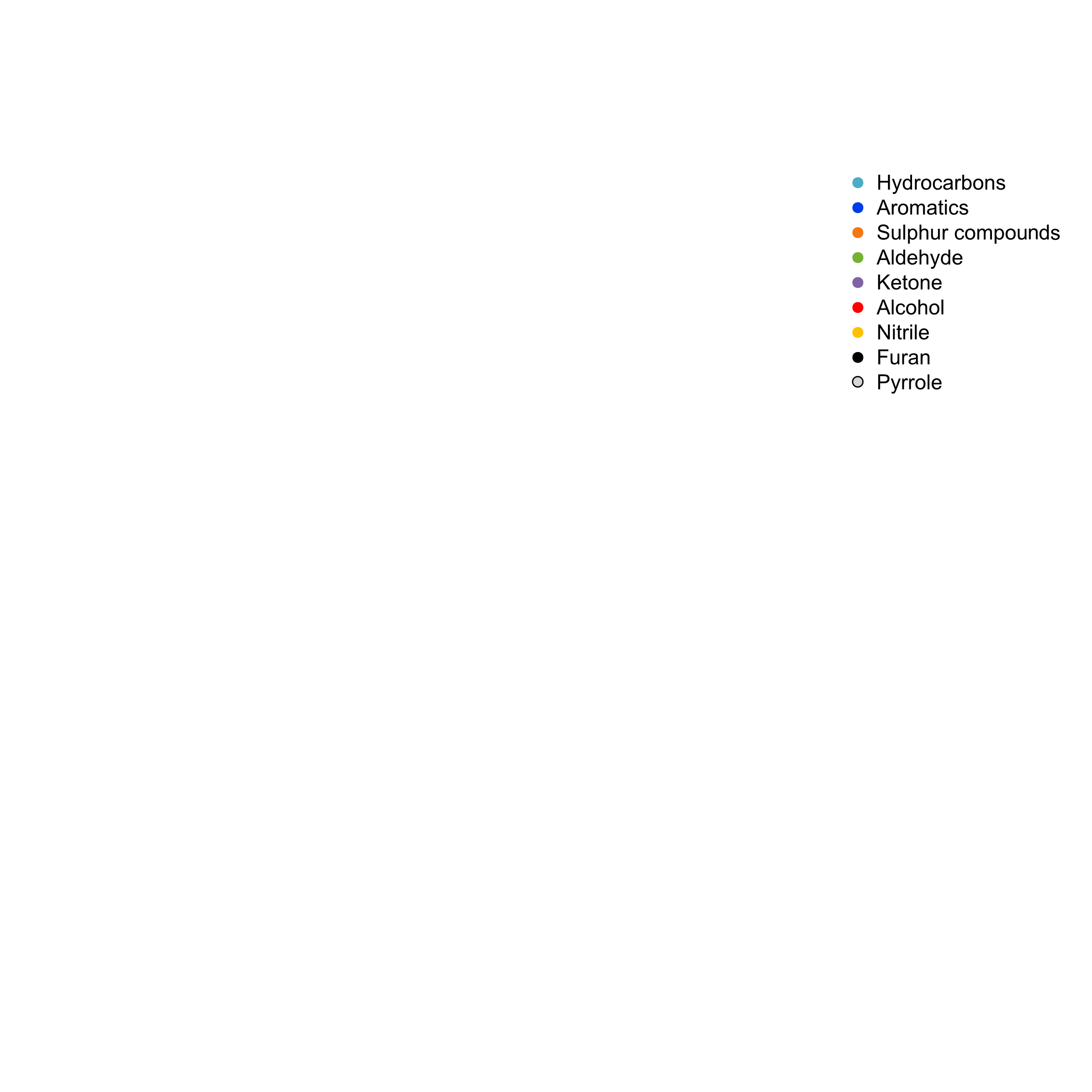
 Aromatic **17**: Benzene; **18**: Benzene, 1,3-dimethyl; **19**: m-Xylene; **20**: o-Xylene; **21**: Styrene; **22**: Toluene;


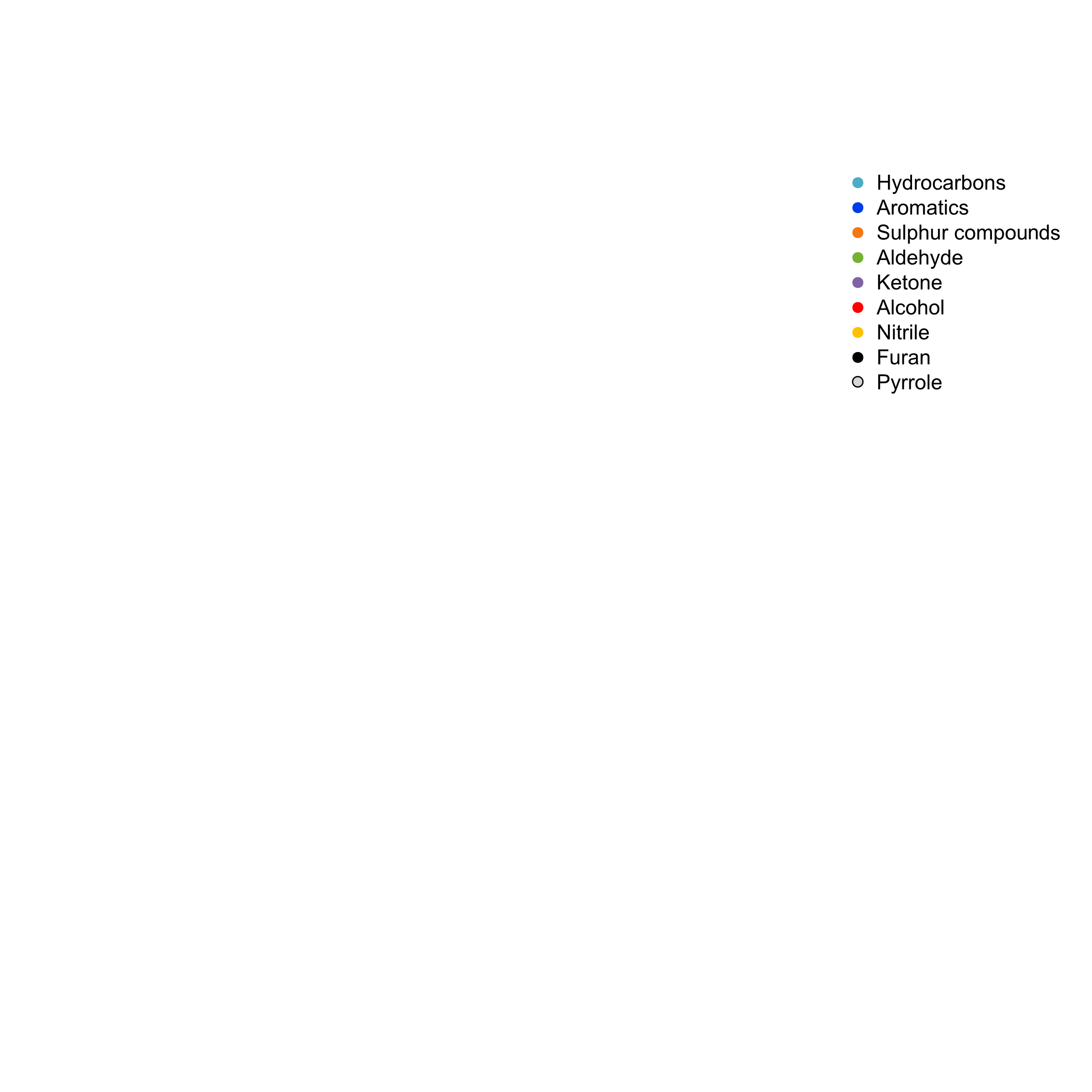
 Furan **23**: Furan; **24**: Furan, 2-ethyl; **25**: Furan, 2-ethyl-5-methyl; **26**: Furan, 2-methyl; **27**: Furan, 2-pentyl; **28**: Furan, 2,3,5-trimethyl; **29**: Furan, 3- methyl; **30**: Furfural;


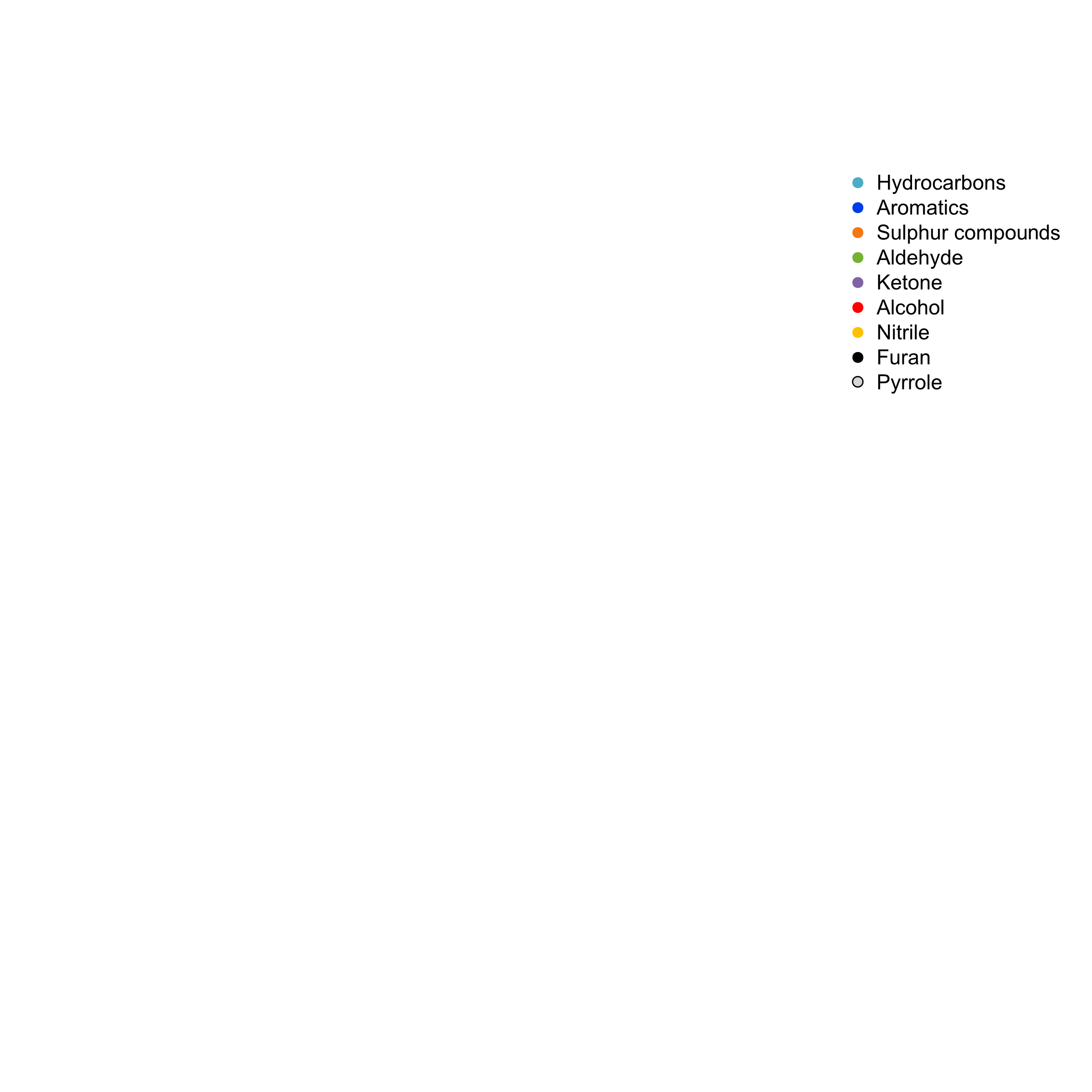

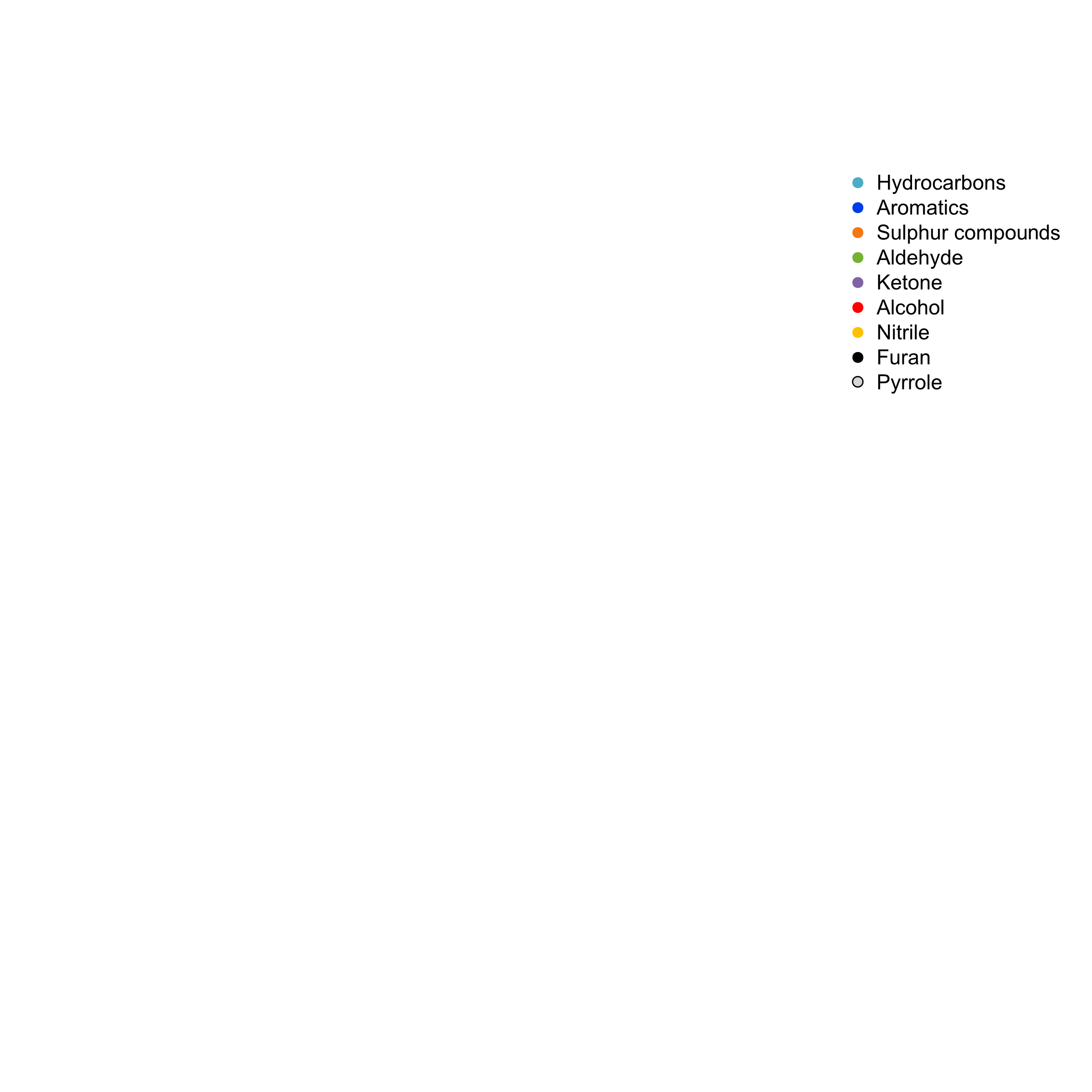
 Hydrocarbon **31**: (Z),(Z)-2,4-Hexadiene; **32**: 1-Ethyl-5-methylcyclopentene; **33**: 1-Hexene, 3-methyl; **34**: 1-Methylcyclohexa-1,3-diene; **35**: 1,3-Cyclopentadiene; **36**: 1,4-Hexadiene, 4-methyl; **37**: 2-Butene; **38**: 2-Hexene, 4-methyl-, (E); **39**: 2-Pentene; **40**: 2-Pentene, 2-methyl; **41**: 3-Heptyne, 5-methyl; **42**: 3,5-Dimethylcyclopentene; **43**: Cyclobutane, (1-methylethylidene); **44**: Cyclopentene; **45**: Cyclopentene, 1-methyl; **46**: Cyclopentene, 3-ethyl; **47**: Pentane; **48**: Propene;


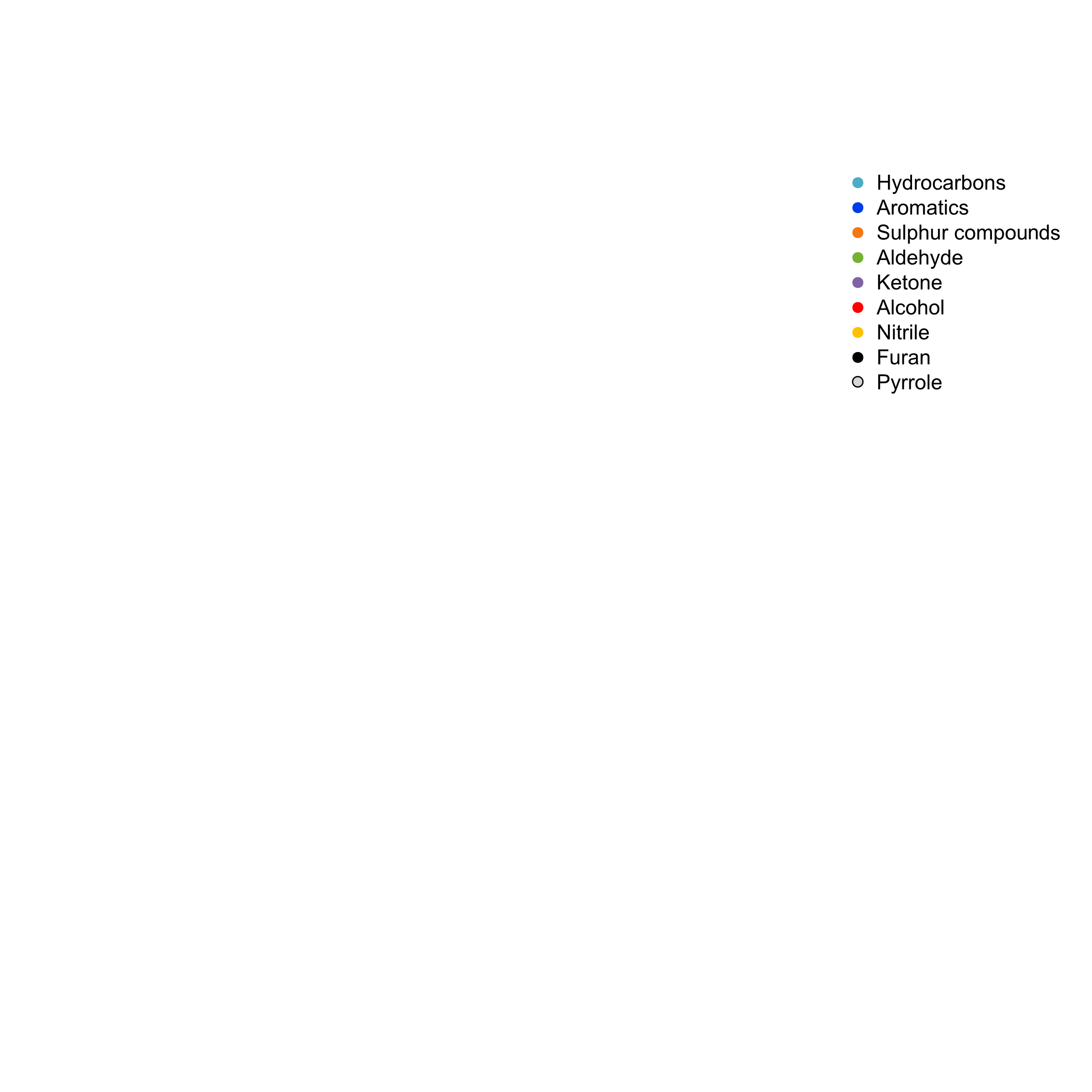
 Ketone **49**: 2-Butanone; **50**: 2-Butanone, 3-methyl; **51**: 2-Heptanone; **52**: 2-Hexanone; **53**: 2-Imidazolidinone; **54**: 3-Pentanone; **55**: 3-Pentanone, 2-methyl; **56**: 4-Heptanone; **57**: Acetone; **58**: Acetophenone, 4-hydroxy; **59**: Hex-4-yn-3-one;


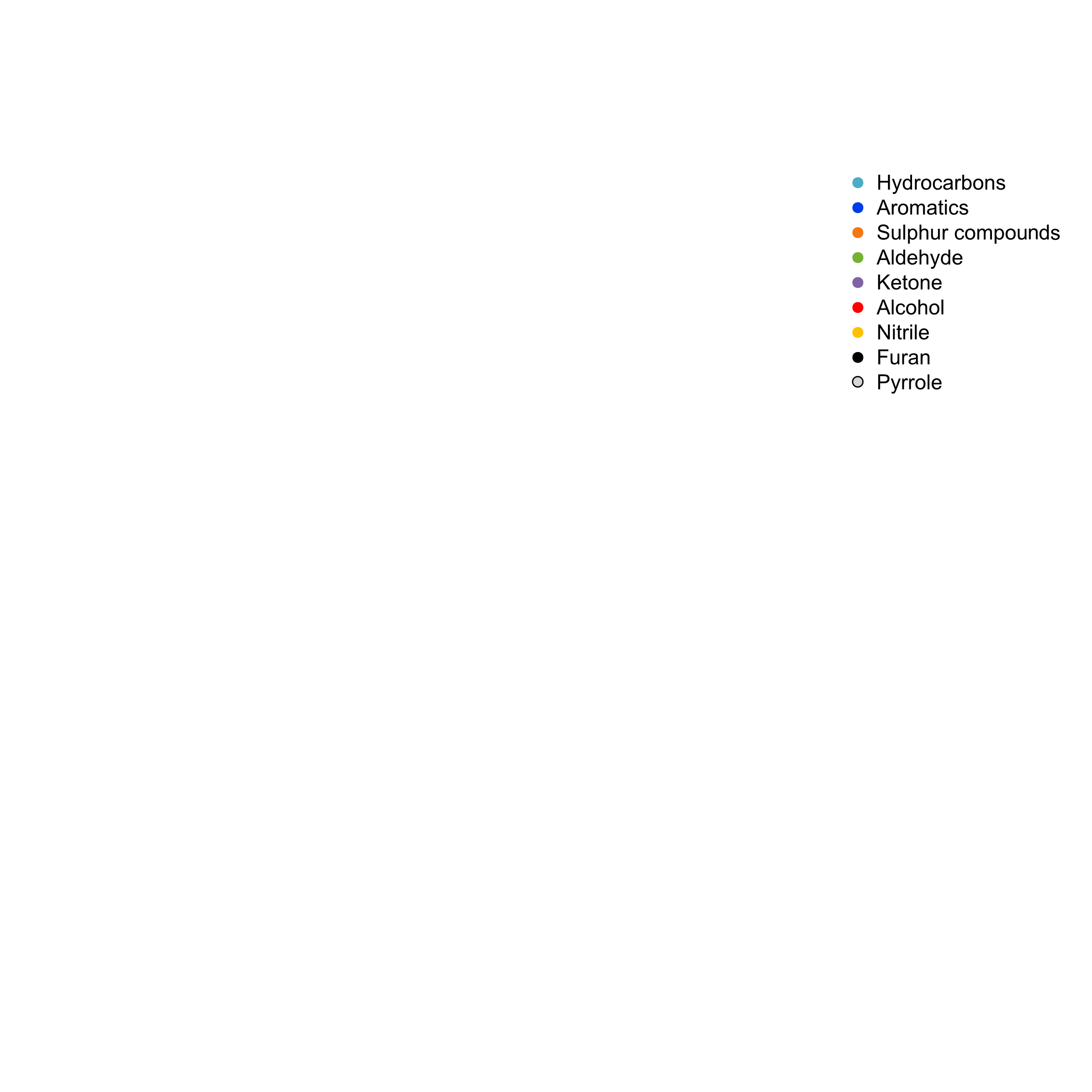
 Nitrile **60**: Isobutynitrile;


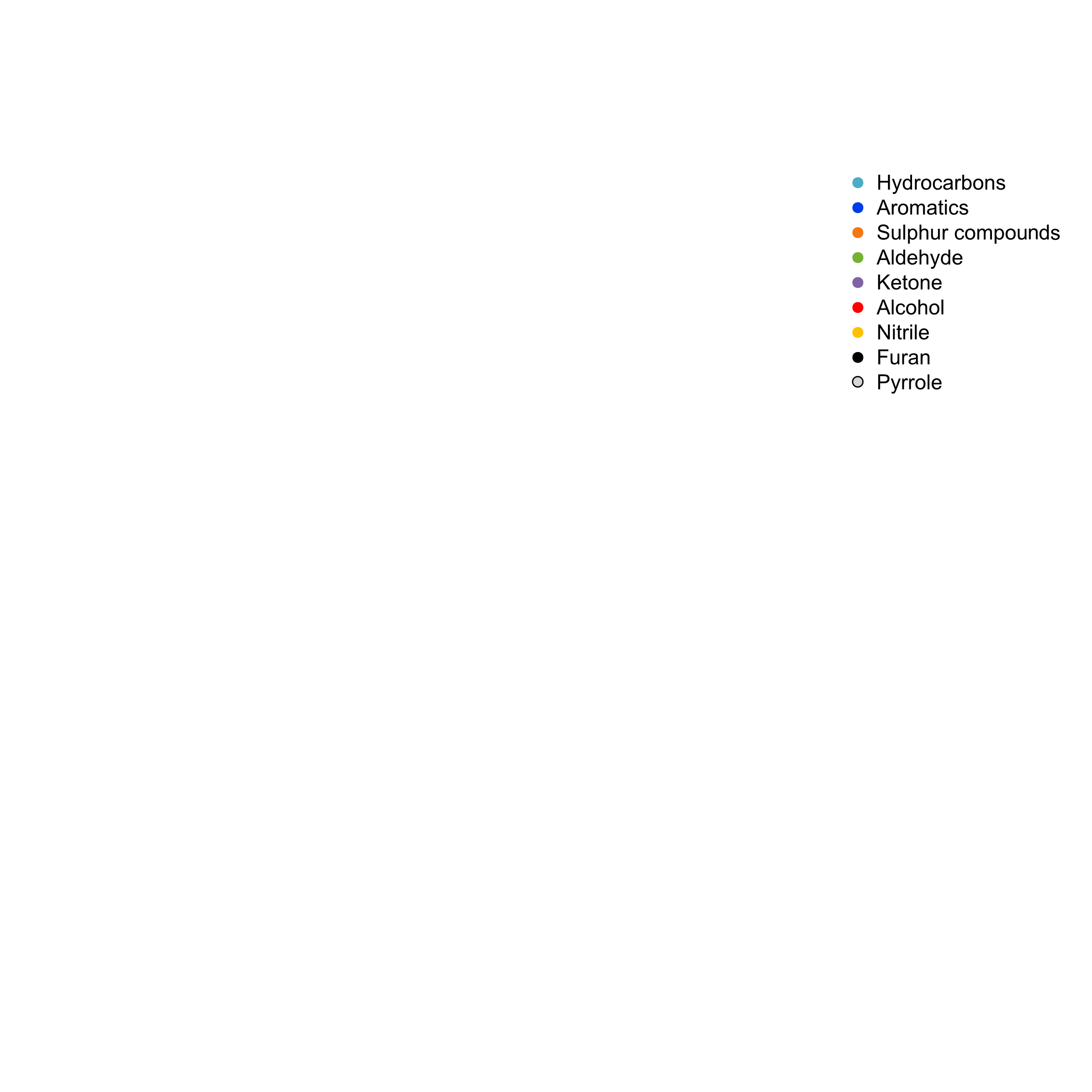
 Pyrrole **61**: 1H-Pyrrole-2-ethanamine, 1-methyl; **62**: 1H-Pyrrole, 1-methyl; **63**: 1H-Pyrrole, 2-ethyl;

Sulphur compound **64**: Disulfide, dimethyl; **65**: Disulfide, methyl propyl; **66**: Methanethiol; **67**: Methyl ethyl disulphide; **68**: Thiophene, 2-methyl; **69**: Thiophene, 3-methyl;
